# Multimodal intelligent biosensors framework for fall disease detection and healthcare monitoring

**DOI:** 10.3389/fbioe.2025.1544968

**Published:** 2025-06-13

**Authors:** Iqra Aijaz Abro, Shuaa S. Alharbi, Naif S. Alshammari, Asaad Algarni, Nouf Abdullah Almujally, Ahmad Jalal, Hui Liu

**Affiliations:** ^1^ Faculty of Computer Science and AI, Air University, Islamabad, Pakistan; ^2^ Department of Information Technology, College of Computer, Qassim University, Buraydah, Saudi Arabia; ^3^ Department of Computer Sciences, College of Computer and Information Sciences, Majmaah University, Majmaah, Saudi Arabia; ^4^ Department of Computer Sciences, Faculty of Computing and Information Technology, Northern Border University, Rafha, Saudi Arabia; ^5^ Department of Information Systems, College of Computer and Information Sciences, Princess Nourah bint Abdulrahman University, Riyadh, Saudi Arabia; ^6^ Department of Computer Science and Engineering, College of Informatics, Korea University, Seoul, Republic of Korea; ^7^ Guodian Nanjing Automation Co., Ltd., Nanjing, China; ^8^ Jiangsu Key Laboratory of Intelligent Medical Image Computing, School of Future Technology, Nanjing University of Information Science and Technology, Nanjing, China; ^9^ Cognitive Systems Lab, University of Bremen, Bremen, Germany

**Keywords:** biosensing devices, artificial intelligence, machine learning, body pose, disease detection, decision-making, healthcare management biosensing devices, healthcare management

## Abstract

**Introduction:**

In the field of human action recognition, the fusion of multi-modal data from RGB and inertial modalities provides a valid technique for identifying activities of daily life and falls.

**Methods:**

Our approach uses two reference datasets: UR-Fall Detection and UMA_Fall Detection for ADL and Fall Events. First, data preprocessing is conducted for each sort of sensor individually, then the signals are windowed and segmented properly. Key features are then extracted, where from RGB data we get 2.5D point clouds, kinetic energy, angles, curve points, ridge features, and inertial signals, giving GCC, GMM, LPCC, and SSCE coefficients. The second method employed is Adam to improve the discriminant of the chosen features. For classification, we employed a Deep Neural Network (DNN) for ADL and fall detection over the UR-Fall dataset and the UMA_Fall dataset.

**Results:**

The classification accuracy achieved on the UMA_Fall dataset is 97% for ADL activities and 96% for fall activities, while for the UR-Fall dataset, it is 94% for ADL activities and 92% for fall activities. This diversified classifier setting compensates for the variety of data and optimizes the system for differentiating between ADL and fall events.

**Discussion:**

The above system provides outstanding results in recognizing these activities on both datasets and illustrates that the multimodal data fusion can boost the human activity identification system for health and safety purposes.

## 1 Introduction

RGB and inertial sensor fusion has become a critical aspect of human action recognition in monitoring precise ADLs and accurately identifying falls (Padha et al., 2023). ADL surveillance also offers valuable functional health information, whereby mobility shifts suggesting impaired health, poor balance, or a higher risk of falling can be identified at an early stage (Das, 2024; Kumar et al., 2021; Mekruksavanich and Jitpattanakul, 2024). Falls are a common issue for elderly people, and recently it has been discovered that almost 28%–35% of people who are 64 years or older experience at least one fall every year. This puts a lot of pressure on health systems and raises severe dangers if medical assistance is not sought (Kakara et al., 2023). Falls are detected with the help of wearable devices, and due to progress in technological and sensor systems, alarms are received rapidly without significant cost expenditure (Wang et al., 2023).

Our research further extends these innovations by using the UR-Fall Detection and UMA_Fall Detection datasets with RGB and inertial data to enhance the classification between ADLs and falls. DNNs have been reported to provide reliable predictions on sequential data, particularly those with temporal characteristics, such as activity recognition. For instance (Keskin et al., 2020) employed DNNs to analyze daily gait tasks, including standing, sitting, and walking, using kinematic data obtained from sensors placed on the pelvis and spine. Their work demonstrated that DNNs are capable of handling temporal dependencies in sequence-based datasets, making them ideal for tracking postural stress and spinal motion. Similarly, (Fridriksdottir and Bonomi, 2020), used accelerometer data for activity recognition, showcasing the flexibility of DNNs in addressing variations in sensor inputs over time. As the datasets in this study are temporal rather than stationary, the ability of DNNs to model temporal patterns makes them a suitable and efficient choice for this research.

Deep Neural Networks (DNNs) are particularly effective in multimodal sensor fusion tasks, achieving state-of-the-art results in activity recognition, as shown in prior studies (Hossain et al., 2023). To achieve accurate classification, we implemented a DNN model, with both datasets undergoing systematic preprocessing, segmentation, and feature extraction. Key features include 2.5D point clouds, kinetic energy, angles and full-body curve, and full-body ridge. For inertial sensors, we extracted Gammatone Cepstral Coefficients (GCC) and Linear Predictive Cepstral Coefficients (LPCC). The robustness of the DNN is demonstrated by its ability to generalize effectively across datasets with diverse data distributions and feature variations. For example, the classification accuracy on the UMA_Fall dataset is 97% for ADL activities and 96% for fall activities, while on the UR-Fall dataset, it is 94% for ADL activities and 92% for fall activities. These results highlight the system’s effectiveness in addressing contextual nuances between datasets.

To enhance feature discrimination, we applied the Adam optimizer (Afsar et al., 2023), which further refines the DNN’s performance. With the help of this proposed multimodal approach, the accuracy of our system is high for recognizing ADLs and fall events. On the basis of continuous health monitoring and support for elderly care applications, the field of human action recognition is improved.

• This paper presents a novel feature extraction approach that is specific to RGB and inertial data to achieve optimal recognition rate of ADL and falls using Adam and improvement of discriminant analysis of key features comprising of the 2.5D point clouds, kinetic energy, and inertial coefficients.
• For the UR-Fall and UMA_fall dataset, feature-specific DNN classifier for ADL detection and fall detection is used. This classifier diversity provides the highest system reliability when working with different datasets.
• By integrating multimodal data from RGB and inertial sensors, the system captures both visual and motion-based information, offering a more comprehensive analysis of human activities and advancing the capabilities of fall and ADL detection frameworks.
• Preprocessing and segmentation techniques are applied to both RGB and inertial data streams, standardizing the input and reducing noise, which contributes to consistent feature extraction and robust performance across varied sensor orientations and settings.
• The research demonstrates the effectiveness of multimodal sensor fusion in healthcare applications, particularly in continuous health monitoring for elderly care, addressing the dual need for precise ADL monitoring and rapid fall detection.

## 2 Literature review

### 2.1 Wearable healthcare monitoring systems

Real-time monitoring of physiological signals is facilitated by wearable healthcare devices for self-management and earlier diagnosis (Di Nuzzo et al., 2021; Kumar, 2024). Some recent research has built upon this space using miniaturized and low-cost designs. Zhuofu et al. (2023) refer to low-cost sensors in terms of the benefits of monitoring heartbeat and temperature using them, despite highlighting multisensor integration and real-time sensitivity issues. Antony (2024) highlight biosensors’ diagnostic capabilities in the absence of conventional bioreceptors, mentioning data variability and security concerns. Arunkumar et al. (2023) investigate post-intervention care through wearables for expedited discharges and streamlined use of resources. Wu et al. (2024) suggest self-powered wearable devices using a nanogenerator, minimizing battery requirements while constrained by portability. These works point toward hybrid, low-cost and real-time wearable devices.

This system builds upon this trend by combining RGB video and inertial sensors for multimodal fusion to increase reliability in activity recognition and fall detection.

### 2.2 MEMS healthcare monitoring systems

MEMS technology enhances diagnostic and monitoring capabilities due to its compactness and sensitivity (Abdulaziz et al., 2021; Liou, 2019; Haus et al., 2022; Shukla et al., 2022) explore MEMS microcantilever arrays for environmental health monitoring, while (Pothala et al., 2024) highlight the potential for miniaturized and biocompatible devices for cancer diagnostics and neurotransmitter monitoring. Moise et al. (2023) suggest a remote healthcare monitoring system for patients and healthcare professionals. It augments real-time decision-making and relieves hospital burden. Infrastructure reliability and data handling in high-volume environments, however, remain under researched. Padha et al. (2023) discuss wearable smart sensors based on MEMS/NEMS technologies, and particularly those embedded in fabrics for real-time tracking of vital signs. Such sensors promote convenience and portability, although durability, signal integrity, and ease of integration in textiles remain research issues. According to Vaibhavi et al. (2017), the usage of BioMEMS is promising for precision surgery and drug delivery while more investigation is needed for other applications, including those of microneedle patch and stent production. Despite the progress made to demonstrate the utility of MEMS in the field of healthcare, cost factors, infrastructure, and the problem of design constraints continue to pose real challenges in MEMS implementation. (Khan et al., 2021). also, discuss MEMS sensors in pandemics including COVID-19 diagnostics. Based on this application, it is unique to see how MEMS technology can be employed in remote diagnostic and treatment, particularly useful during a healthcare emergency.

In all, these studies validate the healthcare transformation potential of MEMS and nanotech while reflecting existing constraints—complexity of integration, scalability of systems, regulatory adherence, and data security. Closing these gaps is essential for achieving successful deployment of these technologies to healthcare environments.

### 2.3 ECG, EMG, and EEG healthcare monitoring systems

Continuously monitoring biomedical signals by ECG, EMG, and EEG is paramount for neurological and cardiovascular diagnostics (Li et al., 2022). A system based on IoT for continuous monitoring of biosignals using ESP32 is offered by (Chakole et al., 2024; Nsugbe et al., 2023) compare and contrast modalities for surgery, reporting ECG’s high precision. Bhatlawande et al. (2024) explored multimodal fusion for psychiatric rehabilitation use and found fusion to still be intricate. EMG and ECG signals are utilized by Byeon et al. (2022) in secure biometric systems and disease diagnosis using CNN, without inclusion of EEG. Bhasker et al., 2022 offer a wireless bio-signal system using dry electrodes to make it highly portable, at the expense of longevity.

The proposed system significantly extends these efforts by integrating multimodal data fusion techniques, combining RGB and inertial sensor features for a more holistic view of patient activity. This approach addresses the privacy, security, and fusion challenges of single-modal systems, ensuring robust classification across heterogeneous data.

### 2.4 Video healthcare monitoring systems

Healthcare monitoring through video has greatly advanced possibilities in the observation of patients at a distance, in patients’ psychological state, and in the care of elderly people with using both online and offline analysis possibilities (Hadjar et al., 2020; Eswaran et al., 2024; Gabriel et al., 2024) offer an AI-powered video monitoring system for real-time hospital behavior monitoring, fall detection, and role identification. Its precision is accurate, yet its performance is subject to diverse camera configurations and finite dataset variety. Patel and Biradar (2024) attain 88.60% accuracy in recognizing behavior based upon HOG, Optical Flow, and SVM in IP webcam and thermal video, although the specific use within the system of recognizing autistic children restricts scalability. Lavanya et al. (2024) propose facial recognition and voice-aided non-intrusive monitoring for Alzheimer’s patients. In improving comfort, its use of vision-based monitoring can overlook meaningful events. Rani et al. (2024) offer an integrated IoT-AI-ML system for predictive diagnosis and healthcare management, while noting infrastructure requirements and system integration issues. Wang et al. (2024) demonstrate contactless vital sign monitoring using video in ICUs and assisted-living units. Despite their advantages, video-based systems face challenges in precision and data privacy.

While video-based systems are noninferior to direct observation through being non-invasive and continuous, these systems need further enhancement in terms of precise detection and challenges in data confidentiality. The proposed system surpasses these limitations by integrating RGB video data with inertial sensor signals, improving detection accuracy through innovative features like 2.5D point clouds, body ridges, and kinetic energy metrics. This hybrid approach enables precise fall detection and healthcare monitoring, ensuring enhanced privacy and adaptability across diverse healthcare scenarios.

### 2.5 Human gait dynamics and human–exoskeleton interaction

Recent work emphasizes the importance of human-exoskeleton coupling for rehabilitation and mobility. Cheng et al. (2024) propose a nonlinear interaction model using neural networks and GPR for force prediction. Peng et al. (2023) integrate adaptive control with dynamic modeling to minimize tracking errors in exoskeletons. Mosconi et al. (2024) assess EMG responses under varying assistance modes, highlighting the need for personalized control. Yan et al. (2023) use damped-spring models to simulate elastic interaction, optimizing comfort and feedback.

These studies underline the relevance of multimodal dynamics. Our system builds on this by capturing comprehensive body movement using hybrid sensor fusion, applicable to both rehabilitation and assistive technologies.

## 3 Materials and methods

### 3.1 System methodology

**FIGURE 1:**
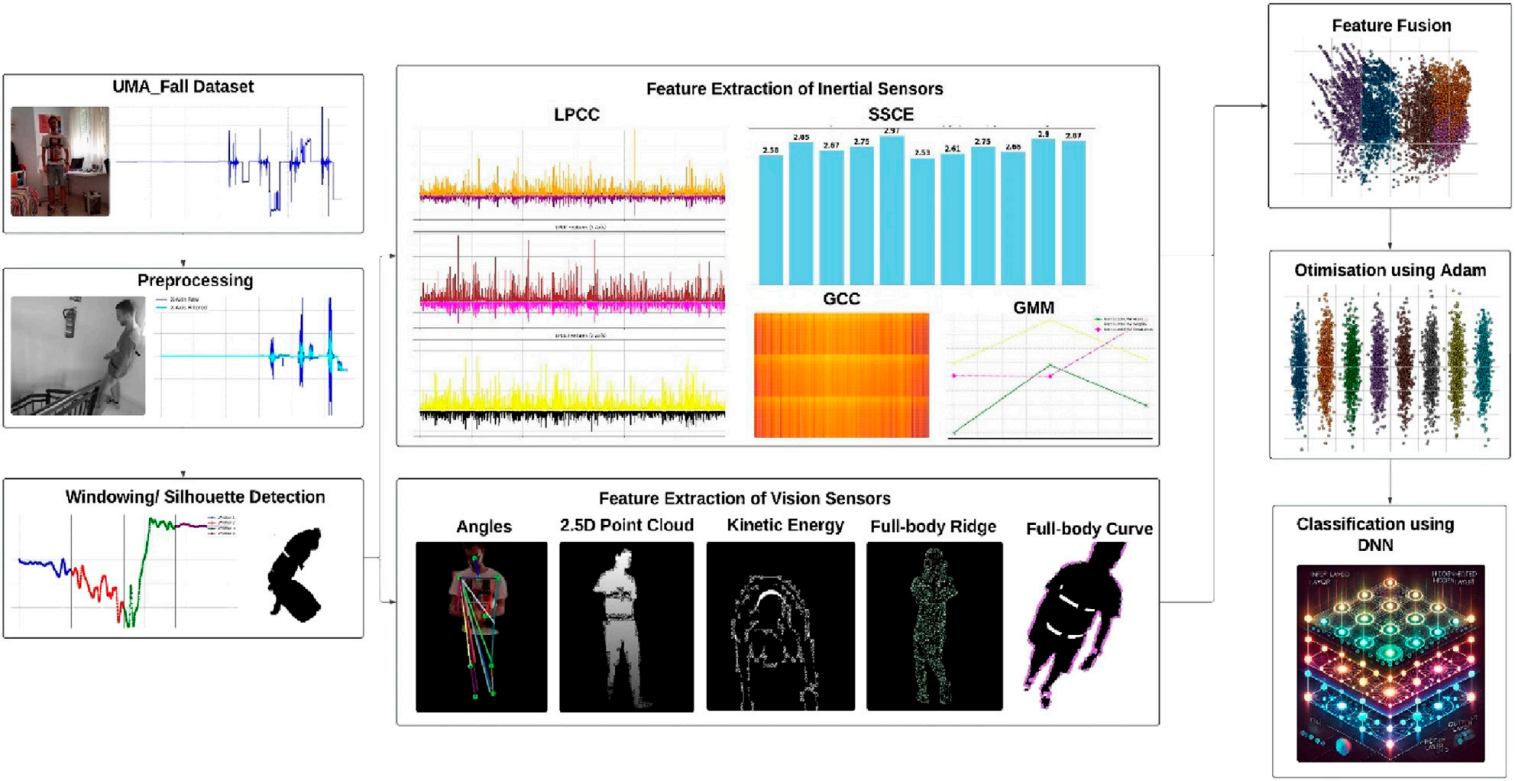
The system architecture of proposed model.

The proposed system processes multimodal data from RGB video and inertial sensors to classify Activities of Daily Living (ADL) and falls, evaluated using UR-Fall and UMA_Fall datasets. RGB data was denoised with a bilateral filter and converted to grayscale for silhouette extraction, while inertial data was filtered using a Kalman filter. Inertial sensors were segmented using Hamming windows for temporal consistency. From RGB data, skeletal keypoints were extracted to compute geometric features like triangles, along with 2.5D point clouds, kinetic energy, and body ridges. Inertial features such as LPCC, GMM, SSCE, and GCC coefficients were derived from windowed signals. Features from both modalities were fused via a common column approach, optimized using the Adam optimizer, and classified using a Deep Neural Network (DNN). The system demonstrated high accuracy in distinguishing ADL and falls, validating the effectiveness of multimodal data fusion. Figure 1 outlines the system architecture used to classify activities of daily living (ADL) and falls by integrating multimodal data from RGB videos and inertial sensors. Each module is represented to demonstrate the preprocessing, feature extraction, fusion, and classification stages.

### 3.2 Sensors filtration and noise removal

This stage involves preprocessing the inertial data with a Kalman filter to enhance the signal and eliminate extraneous noise from the raw accelerometer data. Kalman filter is utilized as best estimator which decreases the mean square error and delivers the present status working from past observation. This guarantees that the signal parameters in noisy data are well retrieved. The filtering is applied to each axis (X, Y, Z) of the accelerometer data, as specified by the state-space model (Equation 1):

$$x_{k}=A\cdot x_{k-1}+w_{k},z_{k}=H\cdot x_{k}+v_{k}$$ (1)

**FIGURE 2:**
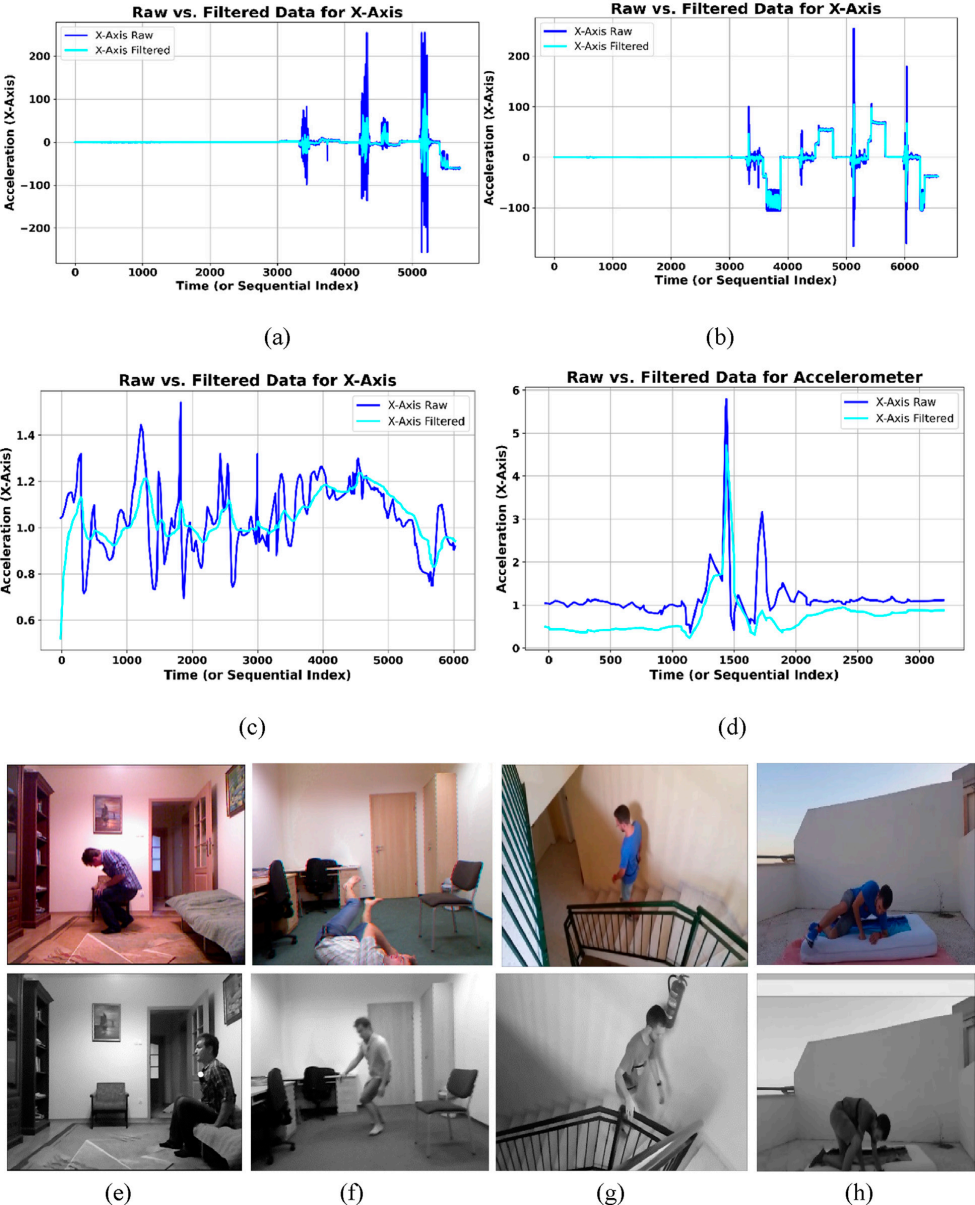
Subplots **(a,b)** show the raw vs. filtered inertial signals for ADL and fall activities in the UMA_Fall dataset, while **(c,d)** depict the same for the UR-Fall dataset. Subplots **(e,f)** present original vs. filtered RGB frames of ADL and fall activities in the UR-Fall dataset, and **(g,h)** show the same for the UMA_Fall dataset.

In this context, $x_{k}$ denotes the current state of the signal, $z_{k}$ measured observation at time *k*, A represents the state transition matrix (how the system evolves), H indicates the observation matrix (how we observe the state), and $w_{k}$ and $v_{k}$ correspond to process and observation noise (random variations), respectively. Filtered signals for each axis are subsequently stored for further analysis. Figures 2a,b depict the raw and filtered inertial signals for ADL and fall activities over the UMA_Fall dataset, respectively. A similar representation is shown for the UR-Fall dataset in Figures 2c,d for ADL and fall activities, respectively. The Kalman filter effectively suppresses noise while preserving critical signal characteristics, enhancing the clarity of activity transitions.

For visual sensor data, the system processes the video stream into individual frames for analysis. All subsequent calculations rely on individual images, not the entire video, necessitating this step. First, RGB frames were converted into grayscaled frames and then bilateral filter preprocess the frames to eliminate noise, thereby facilitating further analyses. The bilateral filter is a non-linear picture smoothing filter that keeps edge information by giving pixels’ different weights based on how far apart they are and how bright they are. We can mathematically represent the bilateral filter as shown in Equation 2.

$$BF\left[I_{x}\right]=\frac{1}{W_{x}}\sum_{y\in S}G_{\sigma d}\left(\left|\left|x-y\right|\right|\right)G_{\sigma r}\left(\left|I_{x}-I_{y}\right|\right)I_{y}$$ (2)

Where, $I_{x}$ represents the filtered intensity value at pixel x (the pixel being processed), $I_{y}$ is the intensity value at neighbouring pixel y , $G_{\sigma d}\left(\left|x-y\right|\right)$ denotes spatial gaussian that gives more weight to nearby pixels, and $G_{\sigma r}\left(\left|I_{x}-I_{y}\right|\right)$ range gaussian that gives more weight to pixels with similar intensities, respectively, σd determines this neighborhood size σr defines the edge amplitude threshold, and $W_{x}$ is a normalization factor to keep the intensity values within bounds. *S* is the set of pixels in the neighbouring around *x*. Figures 2e,f, [Fig F3] illustrate the original and filtered RGB frames for ADL and fall activities in the UR-Fall dataset, respectively, while Figures 2g,h present the same for the UMA_Fall dataset. The bilateral filter significantly reduces noise while maintaining sharp edges, essential for robust silhouette extraction and downstream analysis.

### 3.3 Signal windowing

To segment the continuous sensor data stream into overlapping windows, the Hamming window was used. Moreover, a window size of 100 samples was determined to meet the trade-off between acquiring necessary information of activity in addition to acknowledging realistic constraints on computing magnitude. A smaller window size might not catch vehicle characteristics of the activity or other key happenings, but a bigger size might overwhelm the system with computing expenses or smoothen transitions between activities. A 50% overlap between consecutive windows, equal to a step size of 50 samples, was utilized to provide smooth transitions and continuity in the signal, a standard approach in windowing techniques to balance signal representation and computational complexity. The Hamming window is mathematically defined in Equation 3.

$$w\left(n\right)=0.54-0.46\cos\left(\frac{2\pi n}{N-1}\right)$$ (3)

**FIGURE 3 F3:**
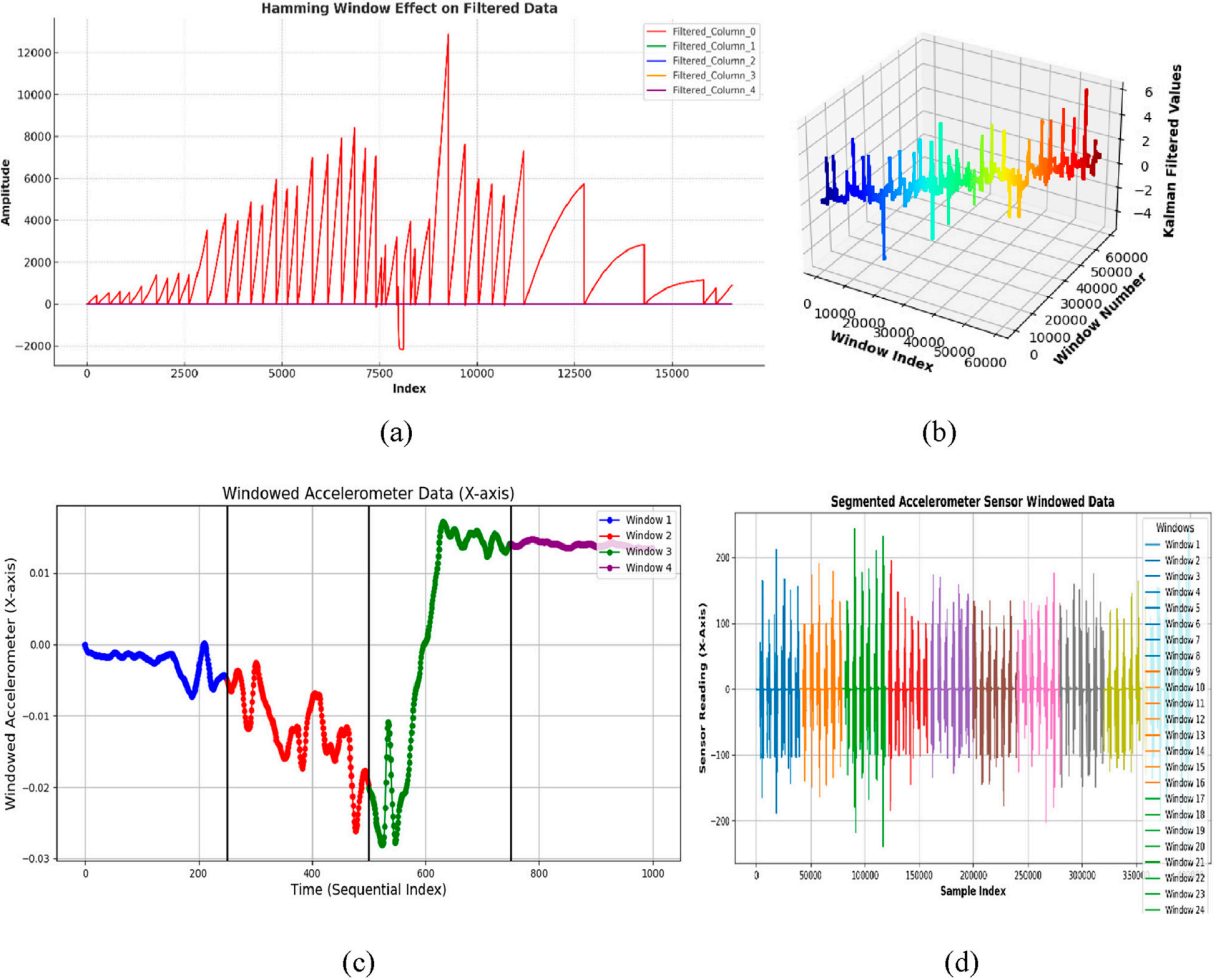
Results of windowed inertial data with **(a,b)** show windowed signal of ADL and fall activities over UR-Fall dataset, respectively **(c,d)** show windowed signal of ADL and fall activities over UMA_Fall dataset, respectively.

Where, $w_{n}$ represents the window weight at sample index *n*, N is the total number of samples in the window. This reduces artifacts caused by sudden edges in the signal. Figures 3a,b showcase the windowed inertial data for ADL and fall activities in the UR-Fall dataset, respectively and Figures 3c,d show the same for the UMA_Fall dataset. The use of a Hamming window ensures smooth signal segmentation while retaining activity-specific details.

To empirically establish the ideal windowing setup, an ablation study on different window sizes and overlap rates was performed. As Table 1 indicates, applying a 100-sample window with 50% overlap produced the highest F1 scores on both datasets consistently. It effectively allows for proper temporal segmentation of steady ADLs and sudden changes during falls without sacrificing computational tractability.

**TABLE 1:** Internal ablation study: impact of window size and overlap on F1 score.

Window size (samples)	Overlap (%)	UMA_fall F1 score (%)	UR_fall F1 score (%)	Observations
50	25	90.2	88.9	Too short for full activity capture
50	50	91.7	89.8	Slight improvement but still incomplete transitions
100	25	94.3	92.1	Better but misses boundaries
**100 (used)**	**50 (used)**	**96.2**	**94.1**	Best overall performance
100	75	96.3	94.0	Slightly better but increases computational cost
150	50	94.1	91.5	Over-smoothing transitions
150	75	94.6	91.7	High cost, no significant gain

Bold values indicate the window sizes (100, 50) used in the proposed model and the corresponding accuracies (96.2, 94.1) achieved with these settings.

### 3.4 Silhouette detection and skeleton modeling

The silhouette segmentation was performed on preprocessed grayscale frames. First, images were converted to the grayscale images to make minor enhancements on the foreground by reducing noise. For human silhouettes extraction from the foreground, Otsu’s thresholding from the output with a binary inversion was done. This method divides the image into two regions, foreground region also known as silhouette and background using a pixel distinction. The thresholding procedure is mathematically stated in Equation 4.

$$T\left(x,y\right)=\left\{\begin{array}{c} 255,if\;I\left(x,y\right)\leq T_{otsu}\\ 0,if\;I\left(x,y\right)>T_{otsu} \end{array}\right.$$ (4)

**FIGURE 4:**
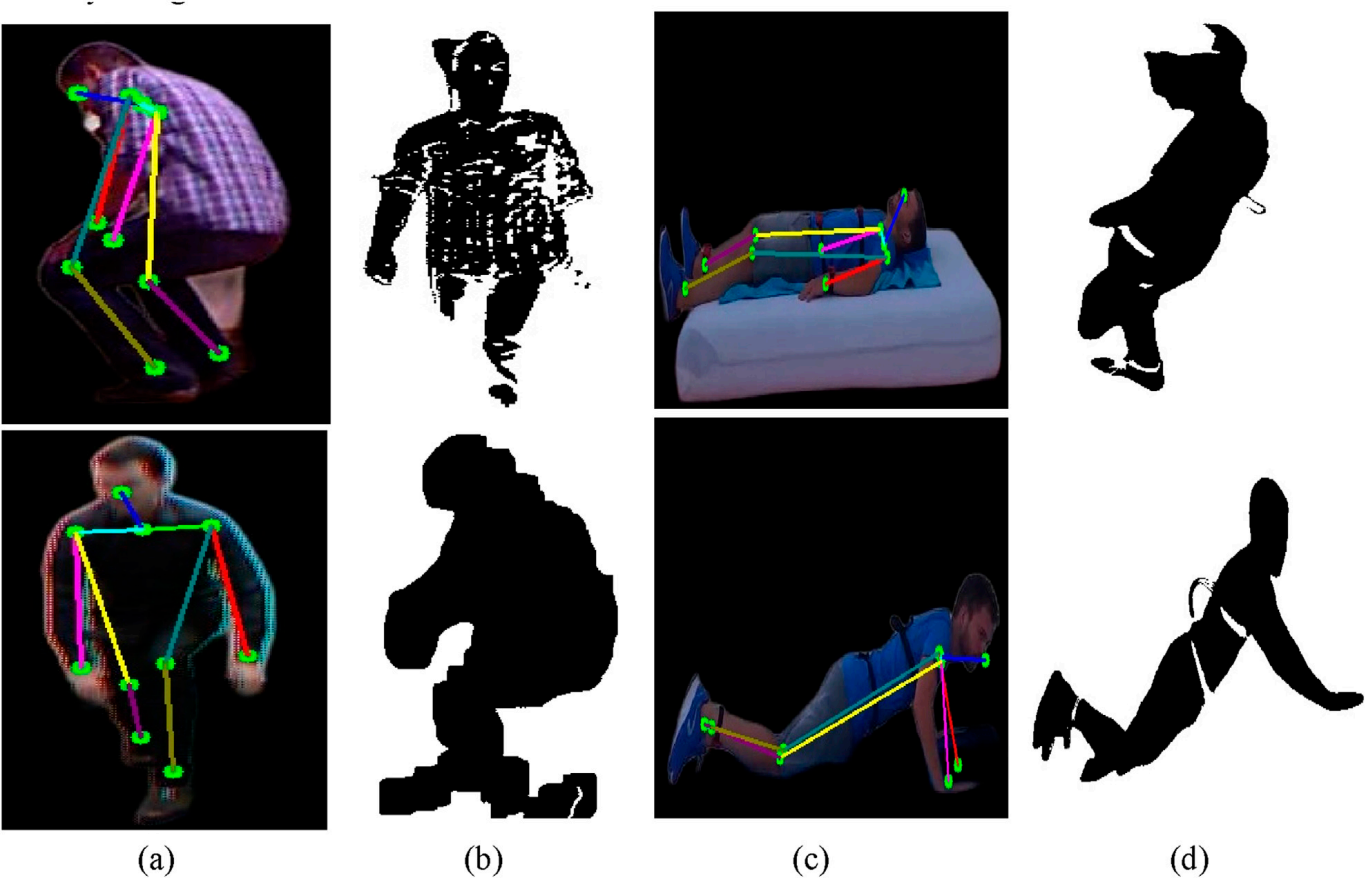
Results of skeleton modeling and silhouette extraction with **(a,b)** show skeleton model and silhouette extraction over ADL and fall activities of UR-Fall dataset, respectively **(c,d)** show skeleton model and silhouette extraction over ADL and fall activities of UMA_fall dataset, respectively.

Where, $T\left(x,y\right)$ represents the output pixel intensity at location $\left(x,y\right)$ , $I\left(x,y\right)$ is the grayscale intensity of the input image, and $T_{otsu}$ is the threshold value determined by otsu’s method. Binary inversion ensures that the silhouette (foreground) is white (255) and the background is black (0). Figures 4a,c show the extracted skeleton models for ADL and fall activities: sitting and falling forward in the UR-Fall dataset, and climbing downstairs and walking in the UMA_Fall dataset. Whereas Figure 4b,d show silhouettes for walking and falling activities in the UR-fall dataset and for climbing down and falling backward in the UMA_fall dataset. The Otsu’s thresholding method effectively isolates the foreground (human silhouette) from the background, resulting in clear binary images for further skeletal modeling.

Moreover, Skeletonization in visual sensor data requires the MediaPipe Pose model (Hsieh, J.-T. et al., 2021) to determine essential body features from RGB frames. The model defines various locations such as the head, shoulders, elbow, hip, and knee that constitute the framework we call a skeletal model. Extra points are earned by finding the mean between consecutive landmarks. For instance, the neck coordinates are determined as the midpoint between the left and right shoulders, as in Equation 5:

$$\left(x_{\text{neck}},y_{\text{neck}}\right)=\left(\frac{x_{L1}+x_{R1}}{2},\frac{y_{L1}+y_{R1}}{2}\right)$$ (5)

Where, $x_{L1},y_{L1}$ are the coordinates of the left shoulder, while $x_{R1,}y_{R1}$ denotes coordinates of the right shoulder. This gives a central neck location based on shoulder symmetry. Similarly, forehead coordinates are determined as the midpoint between the top of the head and face points, as indicated in Equation 6:

$$\left(x_{forehead},y_{forehead}\right)=\left(\frac{x_{H}+x_{F}}{2},y_{H}\right)$$ (6)

Where, $x_{H},y_{H}$ denotes coordinates of the head, while $x_{F},y_{F}$ shows coordinates of the face. Connections between landmarks (e.g., head-to-neck, shoulders-to-hips) are shown with various hues, increasing pose estimation and enabling applications like motion analysis and activity detection. The models include critical body landmarks connected by lines, facilitating detailed motion analysis and activity recognition.

### 3.5 Feature extraction for inertial-based sensor

In the feature extraction phase of our study, we focused on extracting relevant metrics from the UR-Fall Detection and UMA_Fall datasets that effectively represent physiological processes. Specifically, we identified GCC, GMM, LPCC, and SSCE coefficients as essential features due to their robustness in capturing and describing the spectral, statistical, and temporal complexity present in human movement during ADL and fall activities. GCC and LPCC capture the frequency dynamics of sensor signals; GMM models their statistical distributions; and SSCE quantifies movement irregularity. Together, they provide complementary insights into controlled versus abrupt activities, which are critical for real-world fall detection. The selection balances discriminative performance with computational efficiency to enable deployment in practical healthcare environments. However, the framework remains adaptable, and future work may incorporate raw time-series modeling, energy-based descriptors, or deep-learned representations to further enhance classification performance.

#### 3.5.1 Gammatone cepstral coefficients (GCC)

The Gammatone Cepstral Coefficients (GCC) give an enhanced adaptation of standard Mel Frequency Cepstral Coefficients (MFCC), commonly utilized in speech processing. While MFCC efficiently collects low-frequency information, it confronts limitations in dynamic situations and inertial sensor data due to noise sensitivity and limited adaptability. To overcome these restrictions, the GCC is offered in terms of cubic rather than logarithmic operations and employs Gammatone filters in the place of triangular ones, so it can be suitable for using human action recognition employing inertial information. At the beginning of our technique, the signal is partitioned into overlapping frames. We acquire the frequency spectrum using the Fast Fourier Transform (FFT), and then employ the Gammatone filter bank to perform spectral filtering within 26 gammatone filters. A cubical procedure increases the higher frequency component, which shows the fine features of the signal. The GCC coefficients are then transformed using the Discrete Cosine Transform (DCT) for the summed cubic energies, and the classification is robust. Additionally, a logarithmic scaling promotes coefficient interpretability while maintaining sensitivity to complicated manipulations. The GCC coefficients $C_{m}^{\prime }$ are calculated using Equation 7.

$$C_{m}^{\prime }=\log\left(1+\left|C_{m}\right|\right),C_{m}=\sum_{n=0}^{N-1}E_{c}\left[n\right]\cos\left[\frac{\pi m}{N}\left(n+\frac{1}{2}\right)\right]$$ (7)

**FIGURE 5:**
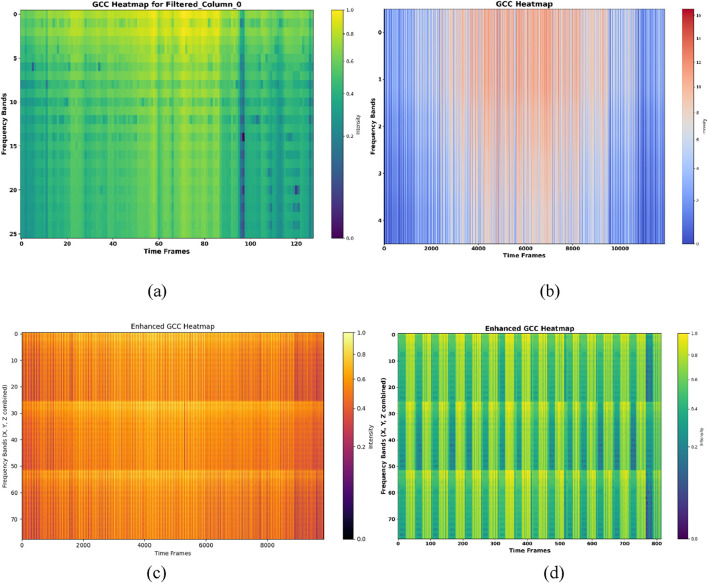
The results for GCC for **(a,b)** show GCC heatmap over ADL and fall activities of UR-fall **(c,d)** show GCC heatmap over ADL activities and fall activities of UMA_fall dataset.

Where, $E_{c}\left[n\right]$ represents the cubic energy output of the $n^{th}$ Gammatone filter $\left(i.e.,E_{c}\left[n\right]={\left.\left|H\right.\left(f_{n}\right)\right|}^{3}\right)$ . $C_{m}$ is the intermediate cepstral value obtained using DCT. *M* denotes index of the cepstral coefficient. *N* shows the total number of filter banks. The logarithmic scaling compresses dynamic range, improving robustness to variations in sensor intensity. Figures 5a,b illustrate GCC heatmaps for ADL and fall activities in the UR-Fall dataset, while Figures 5c,d depict the same for the UMA_Fall dataset. The heatmaps reveal distinct vertical patterns that represent spectral characteristics, enhancing the separability of ADL and falls. This approach provides dynamic feature extraction, employing frequency scaling and complicated transformations for robust signal analysis, as proven in our application to the UMA_Fall and UR-Fall datasets.

#### 3.5.2 Gaussian mixture model (GMM)

Gaussian Mixture Model (GMM) is applied to the preprocessed inertial data to obtain the mean, weight vectors, and covariance of clusters N within the dataset. These parameters give a statistical description of the signal’s underlying structure. The mean vector is calculated using the Maximum Likelihood Estimation (MLE) approach, while the weight vector is computed iteratively to optimize the probability of the model. The covariance performs a type of measure on how far apart the actual signal components have deviated, to gain information on signal variability.

In our method, the combined X, Y, and Z-axis signals are processed through a GMM with *N* = 3 components. The mean (*μ*), weight (*w*), and covariance (Σ) of the GMM components are retrieved as shown in Equation 8.

$$\mu_{i}=\frac{1}{n}\sum_{j=1}^{n}sj,w_{i}=\frac{1}{N}\sum_{j=1}^{n}P\left(s_{j}|i\right),\Sigma_{i}=\frac{1}{n}\sum_{j=1}^{n}{\left(s_{j}-\mu_{i}\right)}^{2}$$ (8)

**FIGURE 6:**
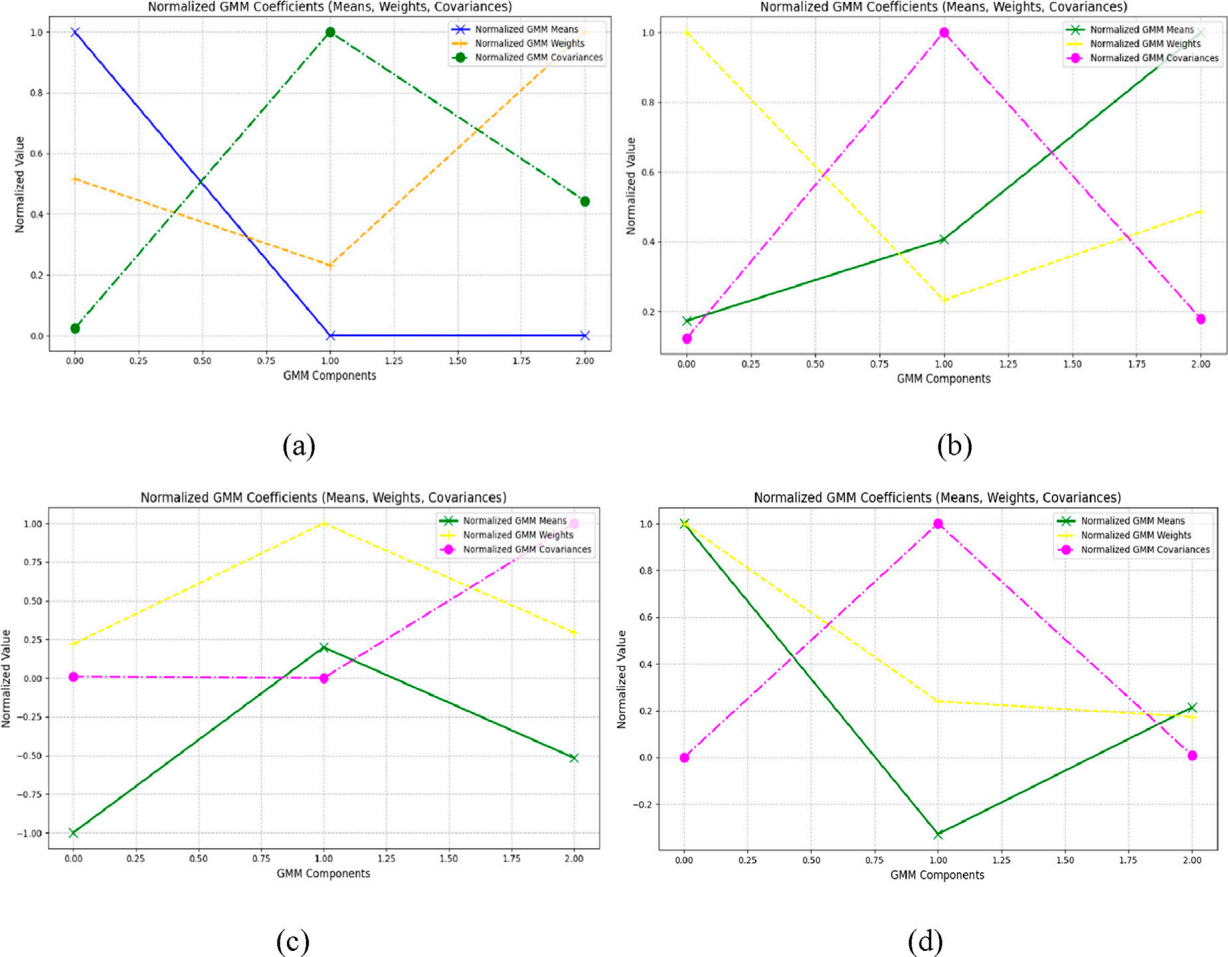
Results of GMM plot **(a,b)** show GMM of ADL and fall activities over UR-fall dataset **(c,d)** show GMM of ADL and fall activities over UMA_fall dataset.

Where, $s_{J}$ is the signal sample, $P\left(s_{j}\left|i\right.\right)$ represents the posterior probability for cluster i , and n is the number of samples. Figures 6a,b visualize the GMM components (mean, weight, and covariance) for ADL and fall activities in the UR-Fall dataset, while Figures 6c,d provide the same for the UMA_Fall dataset. The bar plots highlight the statistical variability captured by the GMM across different activities. These extracted features produced from GMM accurately encapsulate the mean and variance of the inertial data for constructing a stable classification system within the UMA_Fall and UR-Fall datasets.

#### 3.5.3 Linear prediction cepstral coefficients (LPCC)

From inertial signals, Linear Prediction Cepstral Coefficients (LPCC) are generated using the transfer function of the signal and determining the first derivative over frequency bands. The LPCC, in order to reflect signal dynamics, uses a sequence of recursive calculations related with linear prediction coefficients $a\left(x\right)$ .The LPCC coefficients are calculated using Equations 9, 10.

$$c_{x}=a_{x}+\sum_{t=1}^{x-1}\left(\begin{array}{c} x\\ t \end{array}\right)c_{t}a_{x-1,}1\leq x\leq p$$ (9)

$$c_{x}=a_{x}+\sum_{t=1}^{x-1}\left(\begin{array}{c} x\\ t \end{array}\right)c_{t}a_{x-1,}p\leq x\leq d$$ (10)

Where, $c_{x}$ ​ represents the LPCC coefficient, $a_{x}$ ​ is the linear prediction coefficient, *p* is the prediction order, *d* is the total number of coefficients, and $\left(\begin{array}{c} x\\ t \end{array}\right)$ is the binomial coefficient. These equations recursively produce cepstral coefficients for each frame of the signal.

**FIGURE 7:**
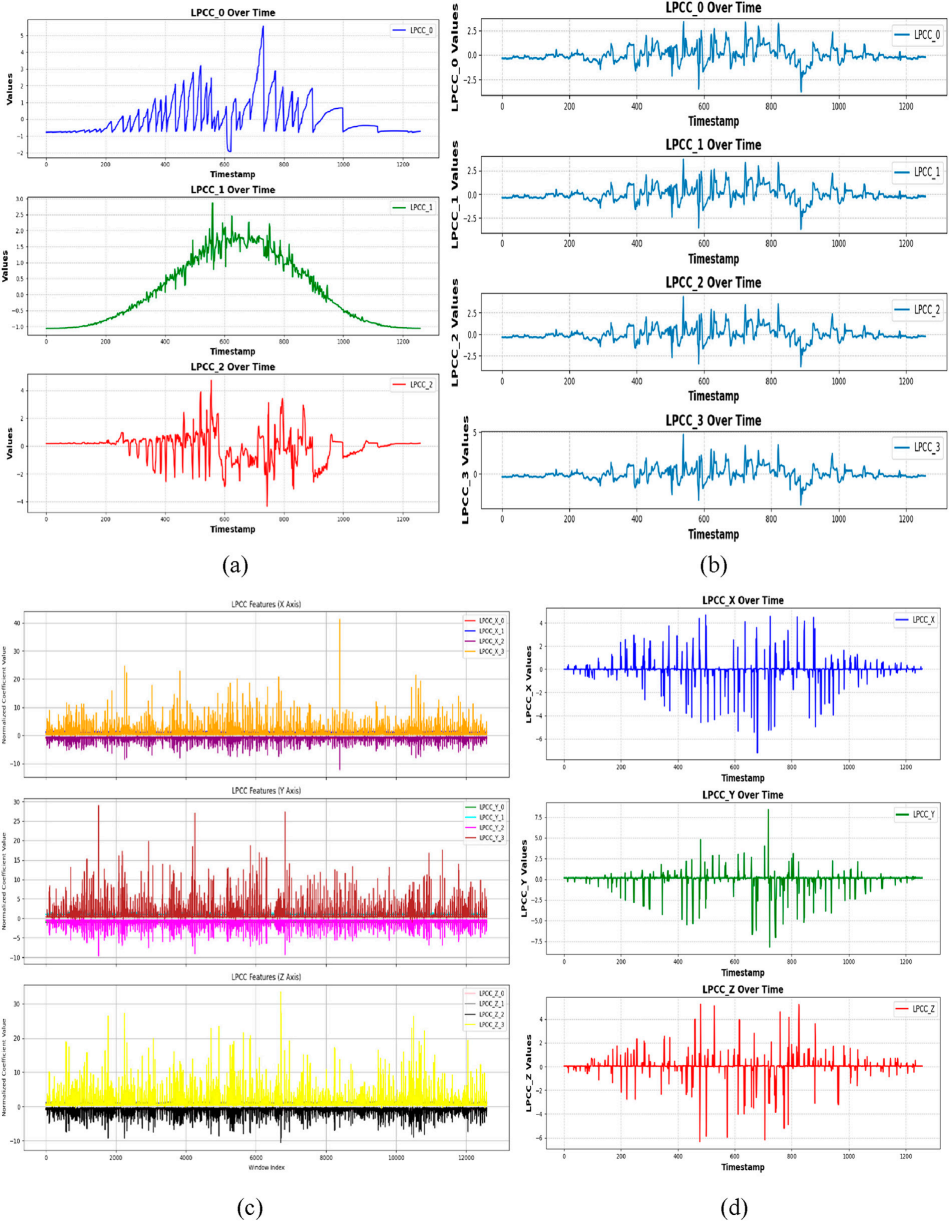
LPCC plotted with **(a,b)** show LPCC plot over ADL and fall activities over UR-fall dataset **(c,d)** show LPCC plot over ADL and fall activities over UMA_fall dataset.

In this work, LPCC coefficients are retrieved for each axis with an order of 12. The estimated LPCC features are shown as time-series plots for the X, Y, and Z-axes, highlighting the temporal fluctuations of the coefficients. This technique offers robust feature extraction, as demonstrated on the UR-fall and UMA_Fall dataset. Figures 7a,b show the temporal variation of LPCC coefficients for ADL and fall activities in the UR-Fall dataset, while Figures 7c,d present the same for the UMA_Fall dataset. The plots reveal distinct patterns: falls exhibit more abrupt and irregular fluctuations compared to the smoother transitions observed in ADL activities. This indicates that falls are characterized by rapid, unstable movements, which is critical for classification.

#### 3.5.4 State space correlation entropy (SSCE)

**FIGURE 8:**
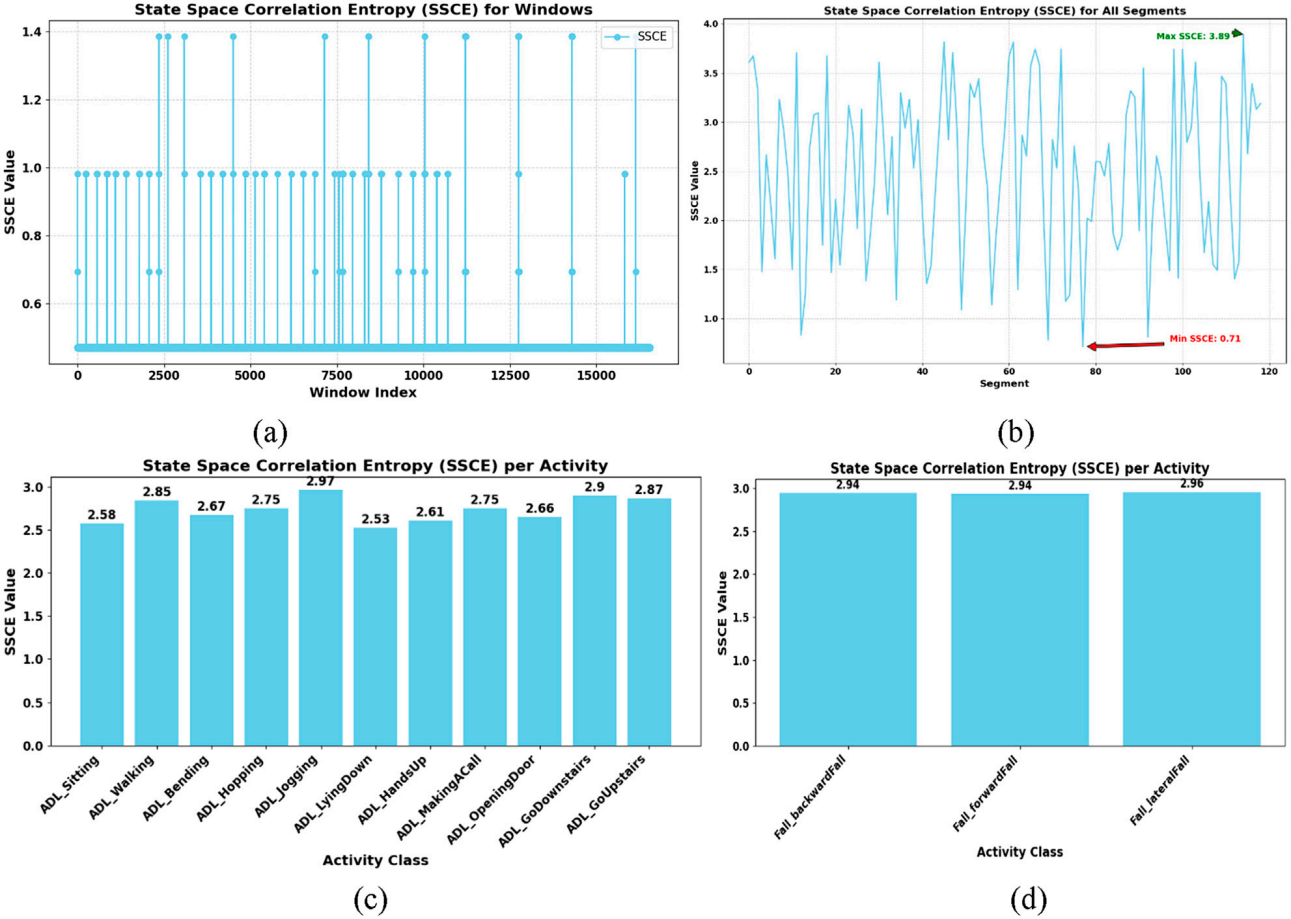
SSCE plotted with **(a,b)** shows SSCE of ADL and fall activities over UR-fall dataset **(c,d)** show SSCE of ADL and fall activities over UMA_fall dataset.

Temporal structure and complexity of signals are examined using SSCE to extract features from physical motion data. The time delay is used to partition the signal into overlapping embedded vectors of the signal. These embedded vectors are then, in a pairwise manner, output in the form of a distance matrix, where diagonal terms represent the correlation of vectors to themselves and off-diagonal terms represent vectors’ distances. We then utilize the covariance matrix from these distances to evaluate the likelihood of correlations, computing the SSCE as given in Equation 11.

$$SSCE=-\log\left(\frac{1}{M^{2}}\sum_{i=1}^{M}\sum_{j=1}^{M}\Theta \left(\epsilon -\left|\left|X_{i}-X_{j}\right|\right|\right)\right)$$ (11)

where $x_{i}$ and $x_{j}$ are the time delay embedded vectors. $\left|\left|X_{i}-X_{j}\right|\right|$ is the euclidean distance, ϵ is a small distance threshold, M is the total number of embedded vectors, and $\Theta \left(x\right)$ is the heavisible step function. The SSCE values are generated for each window and averaged to characterize the complexity of motion signals across activity groups. The results are visualized as bar charts, showing the variability in SSCE values for different activities in the UMA_Fall dataset. This technique provides robust features for discriminating across activity types based on signal dynamics. Figures 8a,b show the SSCE values for ADL and fall activities in the UR-Fall dataset, while Figures 8c,d present the same for the UMA_Fall dataset. The bar charts highlight variability, with falls exhibiting higher SSCE values, indicative of more complex and irregular motion patterns compared to the controlled and repetitive movements of ADL activities. This distinction is crucial for accurately capturing temporal complexity in activity classification.

### 3.6 Feature extraction for vision-based sensor

The feature extraction stage saw us acquire important visual features from the UR-Fall and UMA_Fall datasets. The extracted features—2.5D point clouds, kinetic energy, joint angles, curve points, and full-body ridges—were selected meticulously based on the spatial geometry capture, intensity of motion, direction and quality of body orientation and shape changes necessary to identify ADLs from fall activities clearly. Precisely, 2.5D point clouds capture posture with awareness of depth and hence sudden vertical displacements can be detected. Kinetic energy measures intensity of frame-wise movement and assists with detection of sudden changes. Joint angles provide insight into the coordination and alignment of limbs and curve points and ridges provide capture of deformities in outlines and contours particularly in occlusion or poor illumination scenarios. Collectively, these features play complementary roles synergistically to enable detection in diverse visual and occlusion environments typical to real-world eldercare and healthcare monitoring environments. The current set of features serves the dual purpose of interpretability and computational efficacy; however, the system is extensible and can in the future be extended to include sophisticated descriptors like optical flow, the body’s path-wise metrics, or deep visual embeddings depending on application needs.

#### 3.6.1 2.5D point cloud feature

The resulting 2.5D point cloud comprises both depth, and RGB information to allow exact spatial analysis of motion. From the silhouette images collected from the UMA_Fall dataset, depth values were retrieved and projected onto corresponding RGB frames and then turned into the 3D points. The depth Z was computed using Equation 12.

$$\left[\begin{array}{c} x\\ y\\ z \end{array}\right]=\frac{1}{d}\cdot \left[\begin{array}{c} \frac{\left(u-C_{X}\right)}{F}\\ \left(\frac{{v-C}_{y}}{F}\right)\\ 1 \end{array}\right]\cdot \left(\frac{255}{SF}\cdot \left(1-\frac{v}{H}\right)\right)$$ (12)

**FIGURE 9:**
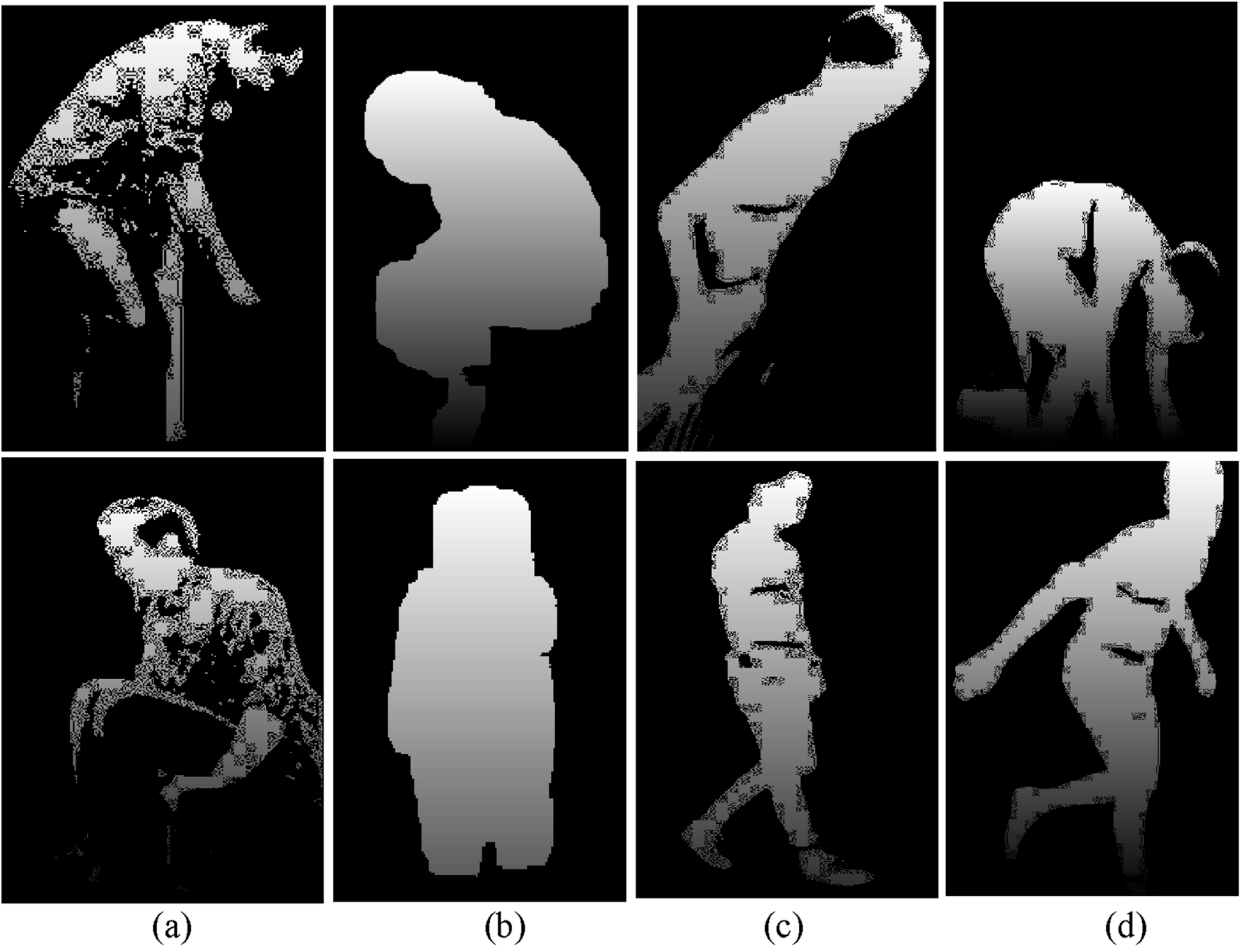
**(a,b)** show 2.5D representations for ADL and fall activities in the UR-Fall dataset, while **(c,d)** display the same for the UMA_Fall dataset.

Here, $\left(u,v\right)$ are pixel coordinates, $\left(C_{x},C_{y}\right)$ are the image center coordinates, F is the focal length. d is the depth scaling factor, SF is the scaling factor, and H represents the image height. This approach integrates depth from silhouettes with RGB pixel data to form point cloud coordinates $\left(X,Y,Z\right)$ effectively modeling body geometry and motion trajectories for robust activity recognition. Figures 9a,b display 2.5D point cloud representations for ADL and fall activities in the UR-Fall dataset, while Figures 9c, d show the same for the UMA_Fall dataset. Falls demonstrate abrupt and uneven distributions of points, reflecting sudden posture changes, whereas ADL activities present smoother and more uniform spatial patterns. This representation effectively integrates spatial and depth information, aiding in robust activity recognition.

#### 3.6.2 Kinetic energy

Kinetic energy preserves the intensity of motion between subsequent frames and acts as an invariant attribute for activity detection. Using the UR-Fall dataset and UMA_fall dataset, for smoothening binary silhouette images of differing motions in successive frames, pixel variation was derived that mirrored the quantitative motion dynamics using Equation 13.

$$KE=\sum_{i=1}^{N}{\left(I_{t}\left(i\right)-I_{t+1}\left(i\right)\right)}^{2}$$ (13)

**FIGURE 10:**
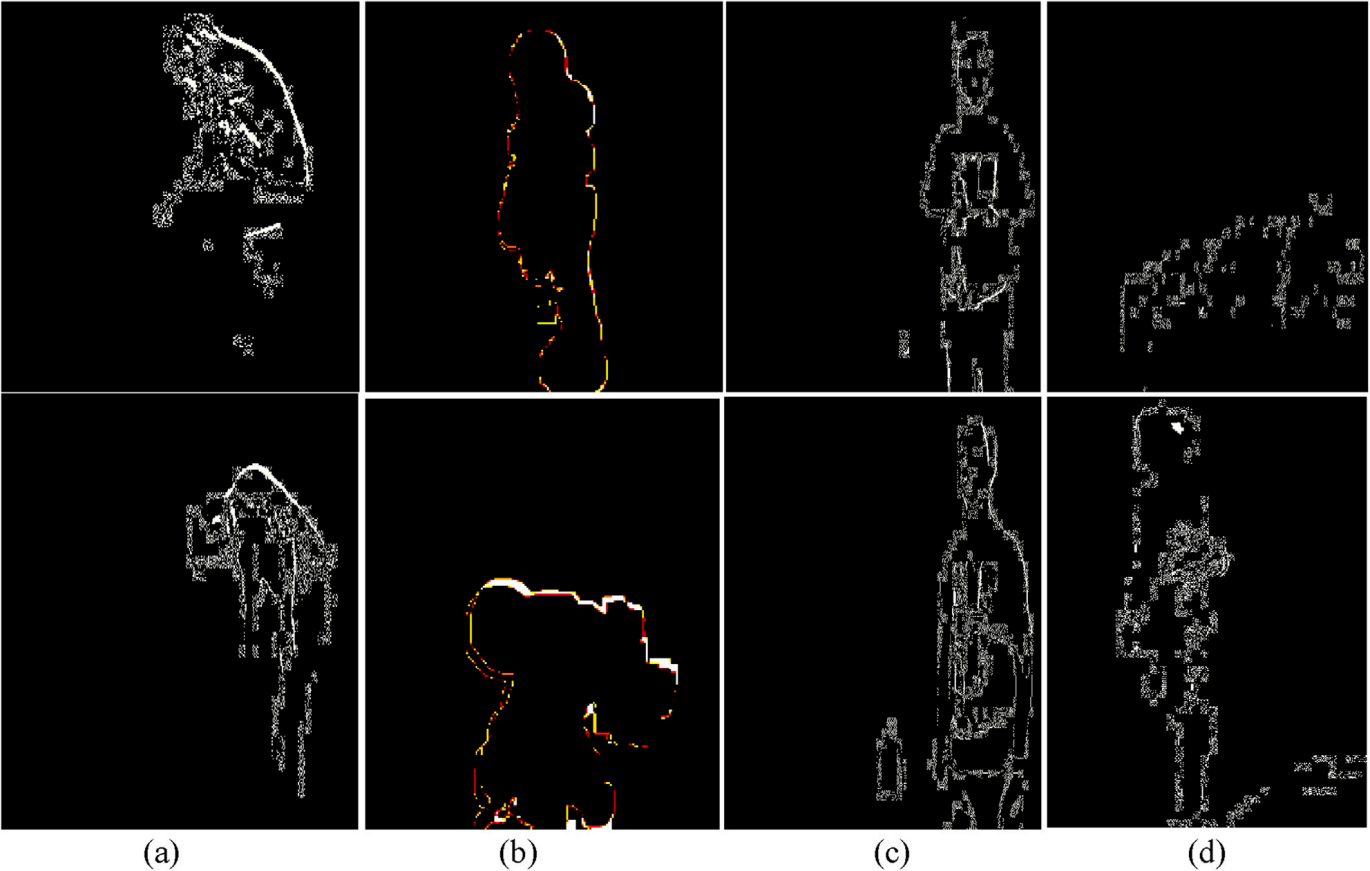
Kinetic energy feature results over various fall and ADL activities from the UR-Fall and UMA_Fall datasets **(a,b)** illustrate kinetic energy variations for ADL and fall activities in the UR-Fall dataset, while **(c,d)** show the same for the UMA_Fall dataset.

Where, $I_{t}$ (i) and $I_{t+1}\left(i\right)$ are pixel intensities at the same location in frames *t* and *t+1*, and N is the total number of pixels in the frame. This equation adds squared pixel intensity differences to describe differences of motion patterns between two successively collected frames. Figures 10a,b illustrate the kinetic energy variations for ADL and fall activities in the UR-Fall dataset, while Figures 10c,d show the same for the UMA_Fall dataset. Falls are characterized by sharp spikes in kinetic energy, corresponding to moments of impact, followed by a rapid decrease. In contrast, ADL activities exhibit smoother energy transitions, representing steady and controlled motion. These variations make kinetic energy a reliable feature for distinguishing between activities.

#### 3.6.3 Geometric feature (triangles)

Joint angles concern the positions of some segments in relation to other segments during particular activities and, as such, supply information regarding dynamic movements. These angles alter based on the activity, thus boosting the action recognition accuracy. The essential body locations allowed twelve joint angles to be determined in this investigation. Each angle was worked out using three pits, and consequently a triangle was involved in the assessment of each angle. The angles were determined with the formula below in Equation 14.

$$\emptyset =arcos\left(\frac{\vec{v_{1}}\cdot \vec{{\;v}_{2}}}{\left|\vec{v_{1}}\right|\left|\vec{v_{2}}\right|}\right)=arcos\left(\frac{\left(x_{B}-x_{A}\right)\left(x_{C}-x_{B}\right)+\left(y_{B}-y_{A}\right)\left(y_{C}-y_{B}\right)}{\sqrt{{\left(x_{B}-x_{A}\right)}^{2}+{\left(y_{B}-y_{A}\right)}^{2}}\sqrt{{\left(x_{C}-x_{B}\right)}^{2}+{\left(y_{C}-y_{B}\right)}^{2}}}\right)$$ (14)

**FIGURE 11:**
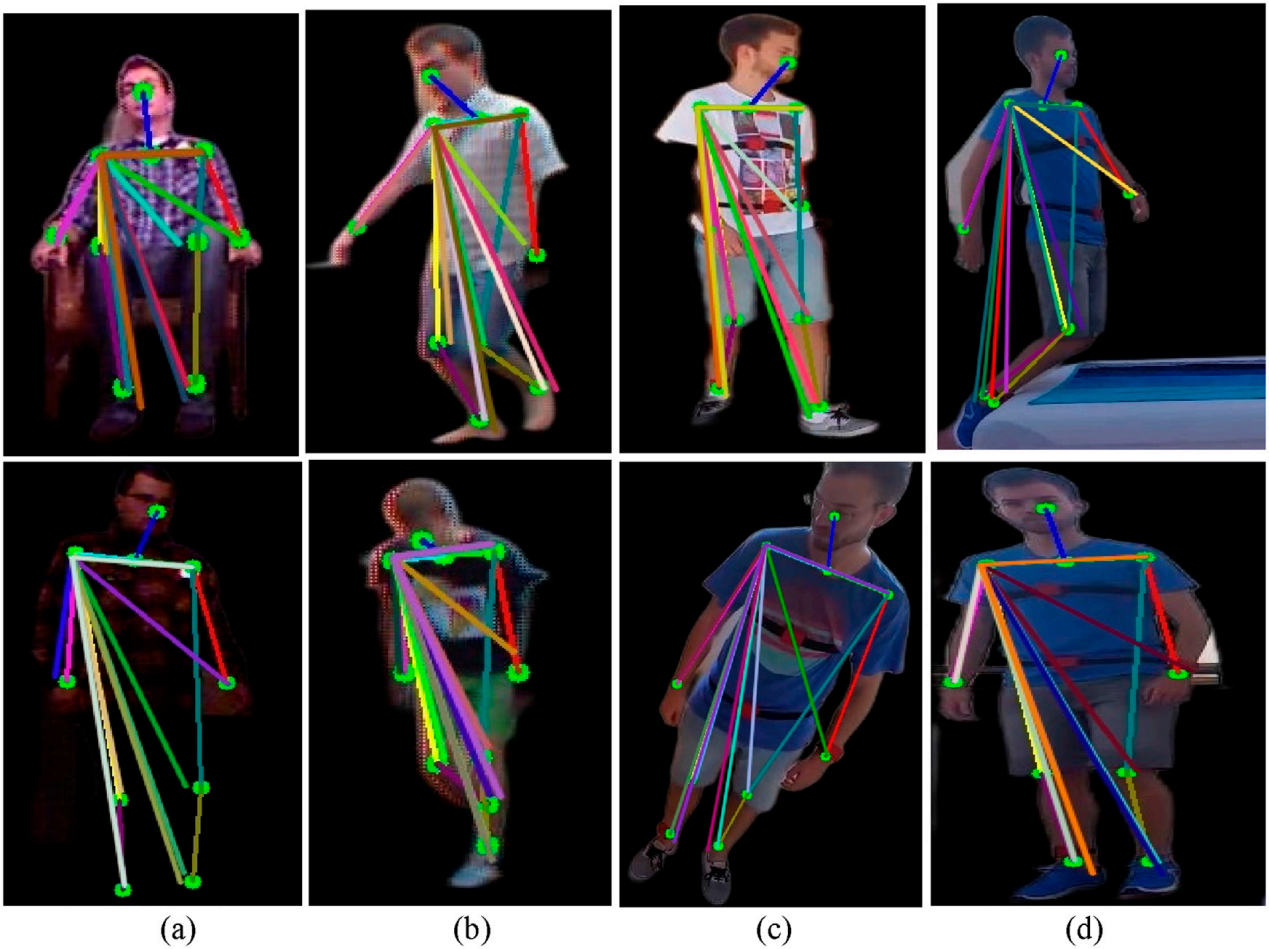
Triangle feature results over various fall and ADL activities from the UR-Fall and UMA_Fall datasets for **(a,b)** depict triangle representations for ADL and fall activities in the UR-Fall dataset, while **(c,d)** show the same for the UMA_Fall dataset.

Here, $\left(x_{A},y_{A}\right),\left(x_{B},y_{B}\right),$ and $\left(x_{C},y_{C}\right.$ ) are the coordinates of the three keypoints. While, $\vec{u_{1}}=\left(x_{B}-x_{A},y_{B}-y_{A}\right)$ and $\vec{u_{2}}=\left(x_{C}-x_{B},y_{C}-y_{B}\right)$ are the vectors connecting the first to the second and the second to the third points. And ∅ is the angle formed at $\left(x_{B},y_{B}\right)$ . This technique guarantees precise tracking of limb direction, facilitating action recognition tasks. Figure 11a,b depict geometric triangles for ADL and fall activities in the UR-Fall dataset, while Figure 11c,d show the same for the UMA_Fall dataset. The angles and shapes of these geometric triangles provide valuable insights into posture and limb dynamics, aiding in accurate activity classification.

#### 3.6.4 Full-body curve

In our research, we achieved this by capturing the complete body contour using canny edge detection and freeman chain coding, which made it easy to recognize the most important contour regions. Canny edge detection enhances the edges of an object, while Freeman’s chain coding codes the object’s contour with a specific directed direction. The utilized eight directional vectors are shown in Equation 15.

$$D=\left[\left(1,0\right),\left(1,1\right),\left(0,1\right),\left(-1,1\right),\left(-1,0\right),\left(-1,-1\right),\left(0,-1\right),\left(1,-1\right)\right]$$ (15)

The silhouette boundary points are specified in Equation 16:

$$C=\left\{P_{0},P_{1},P_{2},\ldots ,P_{n}\right\}$$ (16)

Here, $P_{0}$ is the initial point on the silhouette boundary, and the points proceed in a clockwise direction. Each point $P_{i}$ corresponds to a move in one of the eight directions. Changes in direction along this contour indicate curvature, and are used to detect curve points as represented in Equation 17.

$$P_{c}=\left\{P_{i}|\left(D_{i-1}\neq D_{i}\right)\vee \left(D_{i}\neq D_{i+1}\right)\right\},\forall_{i}\in \left(1,n-1\right)$$ (17)

**FIGURE 12:**
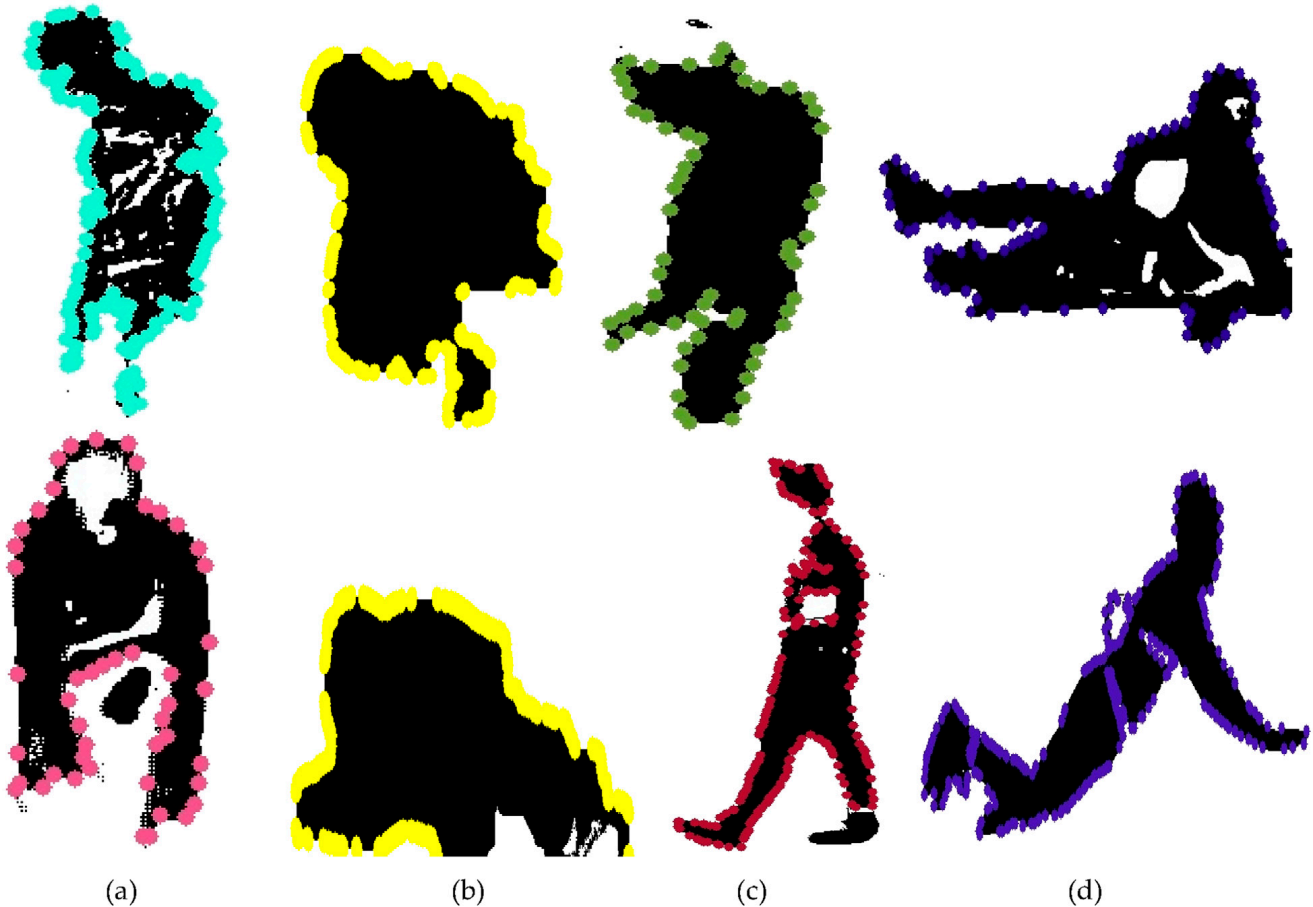
Full-body curve point feature results over various fall and ADL activities from the UR-Fall and UMA_Fall datasets. **(a,b)** show the full-body curve points for ADL and fall activities in the UR-Fall dataset, while **(c,d)** present the same for the UMA_Fall dataset.

The detected curve points were visualized on the original silhouette by marking every *nth* point for clarity. Figures 12a,b show the full-body curve points for ADL and fall activities in the UR-Fall dataset, while Figures 12c,d present the same for the UMA_Fall dataset. ADL activities display evenly distributed curve points, indicating balanced body posture. In falls, the points cluster around areas with rapid contour changes, highlighting irregular postures. This distinction captures dynamic body shapes and supports robust activity recognition. This technique efficiently captures the dynamic properties of the human body’s shape for applications in motion and posture analysis.

#### 3.6.5 Full-body ridge

The ridge characteristics were created from binary edge data acquired using Hessian matrix-based processing. Depth silhouettes were applied to extract binary edges, which were subsequently processed using the Hessian matrix to construct second-order derivatives. Specifically, the matrix components $I_{xx},I_{yy}$ and $I_{xy}$ were computed to detect curvatures in the edges, enabling ridge detection. Ridge response was determined by assessing eigenvalues $\lambda_{1},\lambda_{2}$ of the hessian matrix, isolating pixels where both eigenvalues were negative. These ridges were modeled as interconnecting sequences of pixels, the features of structural nature. The binary edge extraction can be mathematically expressed in Equation 18.

$$R_{ridge}=\left\{\left.p\epsilon I\right|\lambda_{1}<0,\lambda_{2}<0\right\}$$ (18)

**FIGURE 13:**
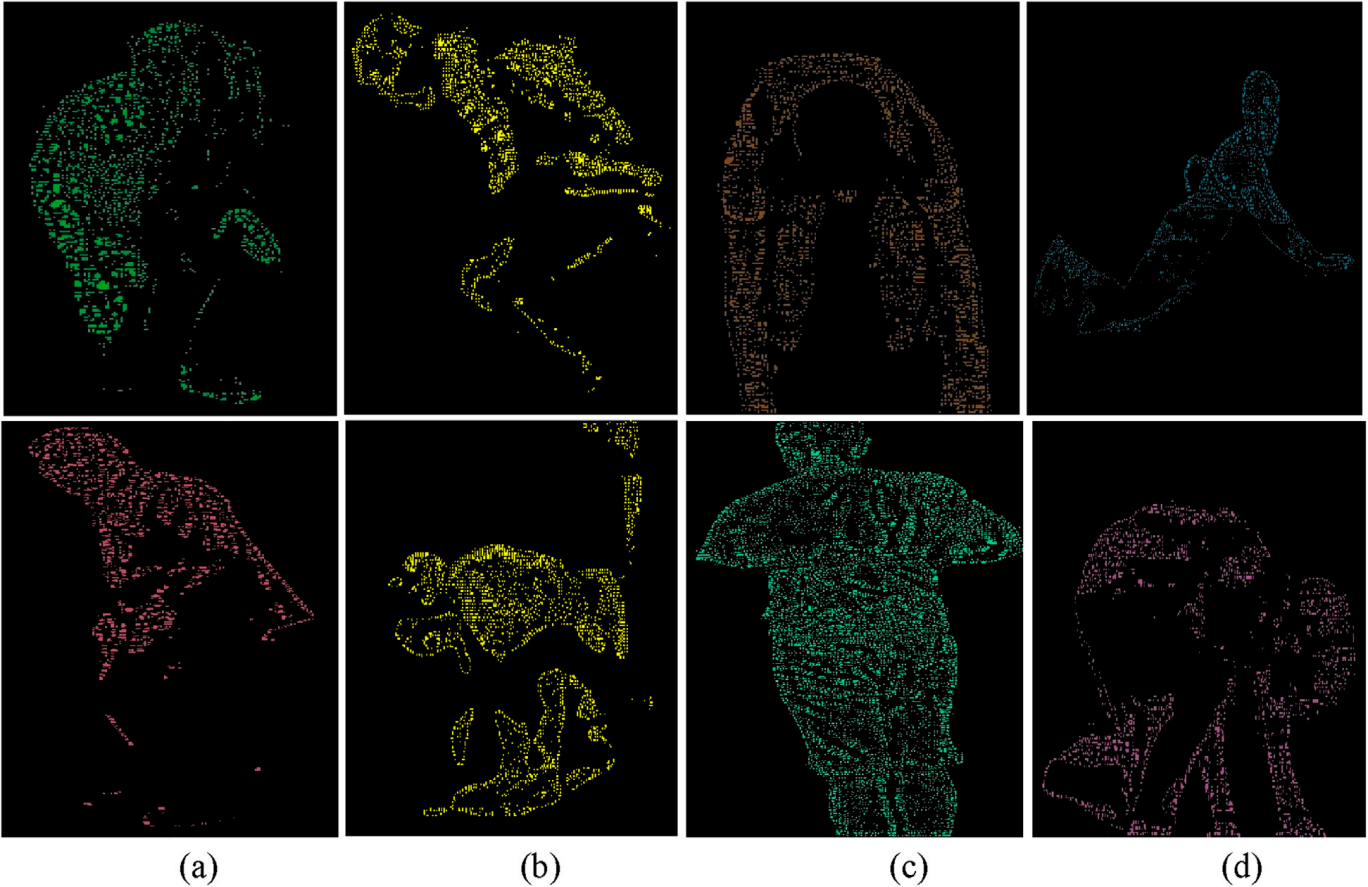
Full-body ridge feature representations with labeled body parts for various ADL and fall activities in **(a,b)** the UR-Fall dataset and **(c,d)** the UMA-Fall dataset.

Where $\lambda_{1},\lambda_{2}$ are eigenvalues of the Hessian matrix when computed from edge pixels. Moreover, for clear differentiation, ridges were arbitrarily colored with random colors. The resulting ridge data $R_{ridge},$ encapsulated within binary edges, approximates skeletal-like features for further analysis. Figures 13a,b present ridge features for ADL and fall activities in the UR-Fall dataset, while Figures 13c,d show the same for the UMA_Fall dataset. Fall activities exhibit sharp and discontinuous ridges, corresponding to sudden and uncoordinated movements. ADL activities, on the other hand, have smoother and more continuous ridges, reflecting stable and coordinated movements. These features effectively capture structural body changes and support activity differentiation.

### 3.7 Feature fusion

In this work, we have used vision-based and inertial-based sensor data to classify falls and Activities of Daily Living (ADL) using a multimodal feature fusion method. Using Hamming windows, inertial properties such as LPCC, GMM, SSCE, and GCC coefficients were first extracted from segmented signals. This process captured both temporal and frequency characteristics. A generalized inertial model was then constructed via intra-modality fusion. Features from the vision-based data, including body ridges, kinetic energy, 2.5D point clouds, and skeletal keypoints, were simultaneously recovered and incorporated into the visual modality. Last but not least, the two modalities were integrated by inter-modality fusion using a well-liked column fusion technique enhanced by the Adam optimizer. The column fusion approach entailed horizontal concatenation of modality features, which were normalized together. All windows were kept consistent in dimensions, and adaptive weights were handled by the DNN model without requiring manually defined fusion weights. To preserve interpretability and modality-specific variance, no explicit dimensionality reduction (e.g., PCA) was applied. Instead, feature selection and optimization were embedded within the dense layers of the DNN, allowing the model to learn modality interactions and eliminate redundancy. This produced a high-level multimodal feature set for ADL and fall classification, which was saved for further use.

### 3.8 Feature optimization

**FIGURE 14:**
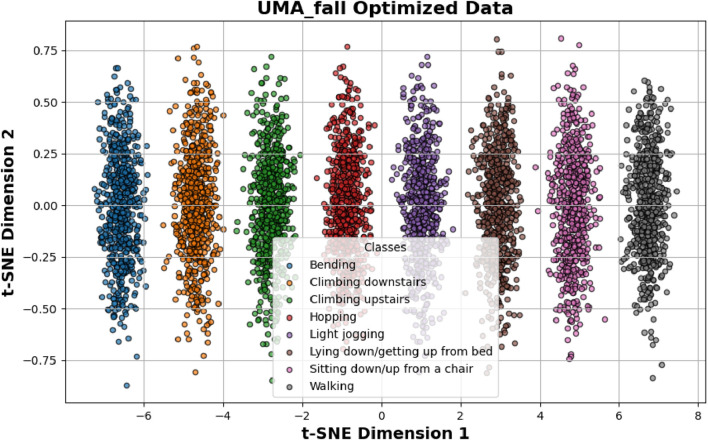
Shows optimization results over UMA_fall dataset.

In this work, we employed the Adaptive Moment Estimation (Adam) optimizer to train a Deep Neural Network (DNN) for the classification of Activities of Daily Living (ADL) and falls. Adam dynamically adjusts the learning rate based on the first and second moments of gradients, computed using Equations 19, 20

$$m_{t}=\beta_{1}m_{t-1}+\left(1-\beta_{1}\right)g_{t}$$ (19)

$$v_{t}=\beta_{2}v_{t-1}+\left(1-\beta_{2}\right)g_{t}^{2}$$ (20)

where $m_{t}$ and $v_{t}$ ​ represent the first moment (mean) and second moment (uncentered variance) estimations, respectively, and $g_{t}$ ​ denotes the gradients at time t . These values are used in adaptive learning rates that produce faster convergence through optimization. Using the Adam optimizer with a learning rate of 0.001 the sparse categorical cross-entropy loss was minimized. The parameters $\beta_{1}$ and $\beta_{2}$ , which determine how quickly the optimizer updates its estimates for the mean and variance of the gradients, were set to $\beta_{1}=0.9$ and $\beta_{2}=0.999$ . These values are widely accepted in modern optimization tasks and have consistently shown strong performance across different machine learning problems (Schmidt et al., 2021; Chavan et al., 2023). To ensure these settings were suitable for the multimodal data in this study, we performed a sensitivity analysis to confirm their stability and effectiveness. Figure 14 illustrates the optimization results for the UMA_Fall dataset. The post-optimization visualization demonstrates improved clustering and feature reparability compared to pre-optimization results. ADL and fall activities form distinct clusters, showcasing the effectiveness of the Adam optimizer in enhancing classification performance by minimizing feature overlaps.

### 3.9 Rationale for choosing deep neural network (DNN) classifier

The choice of Deep Neural Networks (DNNs) for this research was informed by their better ability to learn non-linear and complex patterns in multimodal and temporally dynamic data—abilities basic to our fused RGB and inertial sensor data. In contrast to conventional classifiers like Support Vector Machines (SVMs), Decision Trees, and k-Nearest Neighbors (k-NN), which frequently use manually chosen features and have poor performances in modeling high-dimensional or temporal data, DNNs provide automatic hierarchical feature abstraction suitable for our heterogeneous features such as 2.5D point clouds, kinetic energy, ridges features, and inertial coefficients such as LPCC, GCC, and GMM. Although classical models perform better on low-keyed or static data, their performance breaks down when handling multimodal time-series data due to the inability to model temporal correlations and inter-modality relations basic to human activity recognition tasks. Ensemble models like Random Forests, although good for categorization in general, do not generalize well when feature space is non-homogeneous and temporally inconstant, such as in the cases of ADL and fall events. In contrast, DNNs capture such dynamics intrinsically and performed well in terms of generalization across UR-Fall and UMA_Fall datasets, outperforming other models such as CNN-SVM and CNN-LSTM hybrids documented in earlier research (Modak et al., 2024; Nooyimsai et al., 2022). In addition, integrating the Adam optimizer improved training stability and convergence of DNN, rendering it computationally efficient and highly accurate. In particular, DNNs have been used effectively for similar tasks in human activity identification by using body-worn sensors and video streams, substantiating our selection by drawing upon empirical evidence (Fridriksdottir and Bonomi, 2020; Keskin et al., 2020; Hossain et al., 2023). With such capabilities, DNNs represent an effective and scalable solution for intricate healthcare monitoring platforms, especially those demanding accurate identification of minimal-motion movements and transitions in activities.

### 3.10 Classification

In our research, we used Deep Neural Network (DNN) architecture that is designed to recognize multi-dimensional data points obtained from wearable sensors. This design incorporates several dense layers in the structure of the dropout, which is useful for too many sophisticated sensor data inputs like the accelerometer and gyroscope data. The full architectural configuration of the DNN is detailed in Table 2. Subsequently, each layer performs increasingly pervasively higher-order feature extraction and enables identification of complicated correlations associated with varied activities. The early levels remove recognized and articulated patterns completely and involve just the fundamental intuitive aspects of acceleration, velocity vectors, and direction. It is in this hierarchical structure that it becomes advantageous to Filter for vocations that have similar motion profiles but different contextual subtleties. For this reason, the DNN is adaptable and easy to apply globally with users, especially when other users with a different structure are introduced. Using a wide and balanced set of activities for the training of the network, our architecture produces both accurate and semantically sound categorization results. Specifically, the classification accuracy for UMA_fall ADL activities is 97%, while for UMA_fall fall activities, it is 96%. Additionally, the accuracy for UR-fall ADL activities is 94%, and for UR-fall fall activities, it is 92%. During the training process, such parameters as runtime and memory reveal that the proposed technique works nicely with real datasets. During training, performance metrics such as runtime and memory usage demonstrated that the proposed model handles real-world data efficiently. To further validate the real-time applicability of our model, we evaluated inference latency and memory footprint on a standard computing system: Intel Core i7-10510U (1.80 GHz), 8 GB RAM, Windows 10, using Python in PyCharm without GPU acceleration. The model’s performance demonstrated an average inference latency of about 13.5 milliseconds per instance and maximum memory utilization around 310 MB when evaluated. The outcomes confirm the system to be deployable in real-time or near real-time in healthcare-oriented embedded environments.

**TABLE 2:** Parameter configuration for DNN algorithm.

Parameters name	Values
Initial Learning rate	Dynamic (adjusted by Adam)
Epochs	50
Batch size	32
Dataset split	N-fold cross-validation
Activation function	ReLU (dense layers), softmax (output layer)
Optimizer	Adam

## 4 Performance evaluation

The system in question was examined using two common benchmark datasets. Its performance was thoroughly investigated by confusion matrices, precision and recall metrics, F1 scores, and Receiver Operating Characteristic (ROC) curves, collectively highlighting its usefulness.

### 4.1 Dataset description

#### 4.1.1 UMA_fall dataset

**FIGURE 15:**
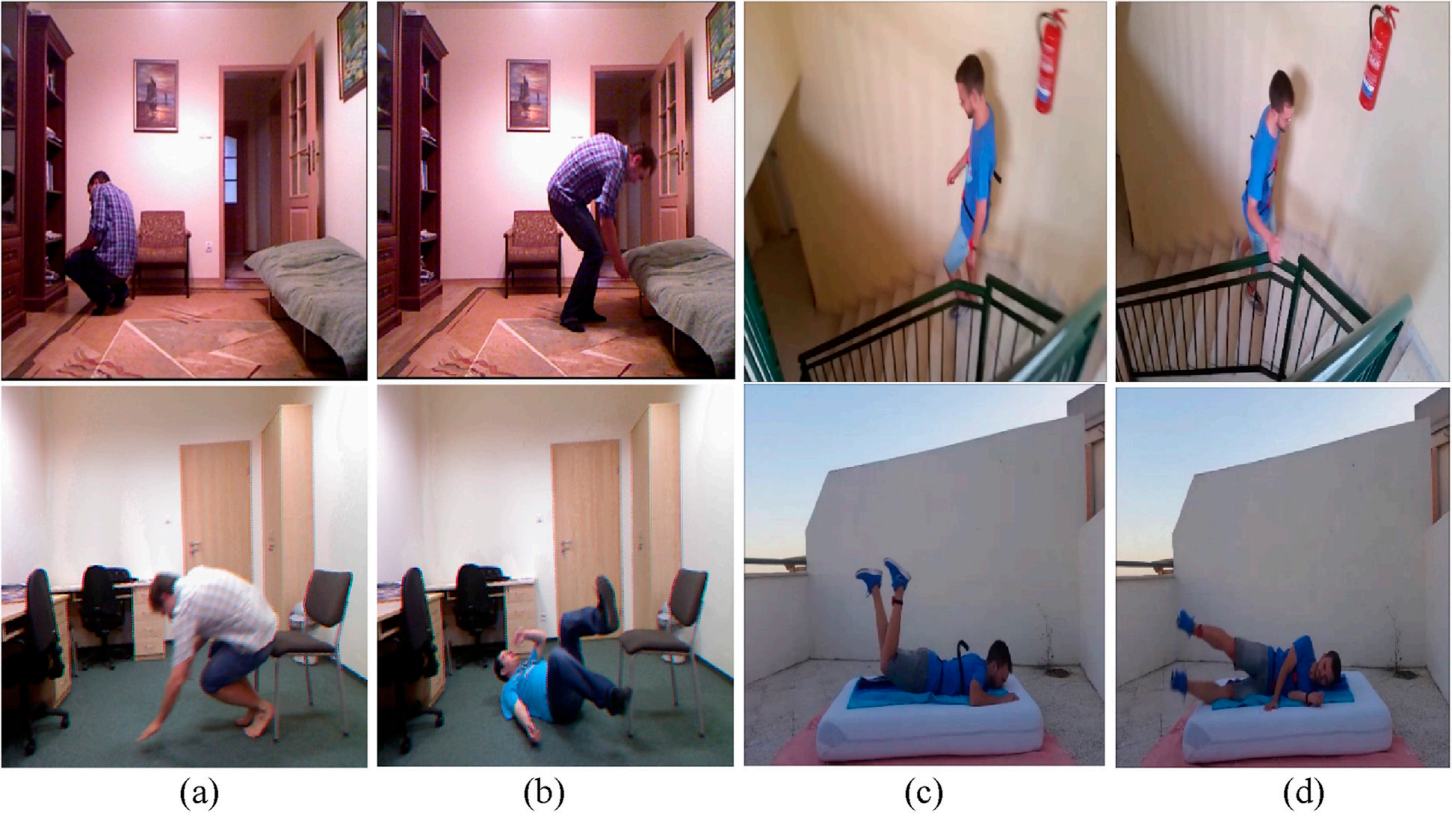
Shows various ADL and fall activities across the UR-Fall and UMA_Fall datasets: **(a,b)** show ADL and fall activities from the UR-Fall dataset, while **(c,d)** show ADL and fall activities from the UMA_Fall dataset.

The UMA_Fall Detection dataset contains data from 19 people aged between 18 and 52 years old; all subjects were of various height, ranging from 155 cm to 195 cm, and weight, ranging from 50 kg to 93 kg. This was done through video participants doing falls and accomplishing ADLs while wearing five internal sensors (gyroscopes, accelerometers, and magnetometers in cellphones) and four external IMUs. The ADLs covered such functions as bending, moving up and down stairs, hopping, mild jogging, reclining and sitting down/getting up from bed/a chair, and walking at a flat rate. Additionally, the dataset covers three fall types: There are mechanical movements, namely,: backward, forward, and lateral. Figures 15c,d represent live examples of dataset.

#### 4.1.2 UR fall detection dataset

The UR Fall Detection dataset was generated by gathering data from two Kinect cameras connected via USB and an IMU device worn on the waist and paired via Bluetooth. Activities of Daily Living (ADLs) were collected using camera 0 and an accelerometer, while devices such as the PS Move and x-IMU captured additional sensor data. Five participants participated, undertaking 70 sequences that included 30 falls and 40 ADLs within an office scenario. Falls were done onto a carpet approximately 2 cm thick, with the x-IMU positioned at the pelvis. Each volunteer conducted forward, backward, and lateral falls at least three times, as well as ADLs such as standing, sitting, squatting, bending, picking up goods, and resting on a sofa, as shown in Figures 15a,b. All deliberate falls were properly detected, with rapid sitting movements categorized as ADLs despite their similarity to falls when evaluated with an accelerometer or a combination of accelerometer and gyroscope. The dataset also includes falls from standing positions and while sitting on a chair. Raw accelerometer data and depth and RGB image sequences collected by two Kinect cameras were kept for each incident. Additionally, a threshold-based fall detection system was implemented, with images obtained using Microsoft Kinect cameras.

Although the proposed system functions reliably on both datasets, there exist data collection-related limitations that should be noted. Firstly, the datasets are predicated on fixed sensor placement, which may not directly translate to practical deployments and could impact signal quality. Secondly, although the UMA_Fall dataset contains participants with diverse height and weight ranges, both datasets are confined to healthy adults and exclude elderly, pediatric, and physically challenged participants to restrict generalizability. Finally, data were acquired under constrained conditions, with real-world environments potentially bringing extraneous issues like sensor movement, occlusion, or unmodeled activities to the fore. Remedying these concerns is critical to future clinical or home-based deployments.

## 5 Results and analysis

In this section, various experiments were conducted to evaluate the proposed system. The evaluation utilized metrics such as the confusion matrix, precision, recall, F1 score, and Receiver Operating Characteristic (ROC) curve. A comprehensive discussion and analysis of the results are provided below.

### 5.1 Experiment 1: confusion matrix

In the first experiment, we plotted the confusion matrix for both datasets. The confusion matrix gives a concise visual representation of the classifier’s performance, emphasizing its strengths and limitations in terms of how it handles different classes. Tables 3 and 4 exhibit the confusion matrix for the UMA_fall ADL and fall activities, respectively. While tables 5 and 6 show confusion matrix for the UR-fall dataset over ADL and fall activities, respectively.

**TABLE 3:** Confusion matrix calculated over the ADL activities of UMA_fall Dataset.

Obj. Classes	BD	CD	CU	HP	JG	LD	SD	WK
BD	97	1	0	0	0	0	1	1
CD	0	97	1	0	0	1	1	0
CU	0	0	97	1	0	0	1	1
HP	0	1	1	96	1	0	0	1
JG	0	1	0	0	97	1	0	1
LD	0	1	1	1	0	96	0	1
SD	1	0	0	0	1	1	97	0
WK	0	0	1	0	1	0	1	97
Mean Accuracy = 97%								

BD , bending; CD , climbing downstairs; CU = climbing upstairs; HP = hopping; JG = light jogging; LD , lying down (and getting up) on (from) a bed; SD , sitting down (and up) on (from) a chair; WK = walking.

**TABLE 4:** Confusion matrix calculated over fall activities of UMA_fall Dataset.

Obj. Classes	Fall backward	Fall forward	Fall forward
Fall backward	95	3	2
Fall forward	2	94	4
Fall lateral	3	2	95
Mean Accuracy = 96%			

**TABLE 5:** Confusion matrix calculated over ADL activities of UR-fall Dataset.

Obj. Classes	ST	SI	LD	BD	CR	WLK	PR	OT
ST	95	2	1	0	0	0	1	1
SI	1	94	1	0	0	2	1	1
LD	0	1	95	2	0	0	1	1
BD	0	2	2	93	2	0	1	0
CR	1	1	0	1	94	1	1	1
WLK	2	1	1	1	0	94	0	1
PR	1	1	1	0	1	1	94	1
OT	1	0	2	1	1	0	1	94
Mean Accuracy = 94%								

ST, standing; SI, sitting; LD, lying down; BD, bending; CR, crawling; WLK, walking; PR, praying; OT, others.

**TABLE 6:** Confusion matrix calculated over fall activities of UR-fall Dataset.

Obj. Classes	Fall forward	Fall backward	Getup (chair)	FWS	FWST
Fall Farward	92	4	2	1	1
Fall Backward	3	91	4	1	1
Getup from (chair)	2	2	92	2	2
FWS	1	2	3	92	2
FWST	1	1	2	3	93
Mean Accuracy = 92%					

FWS, falling when seated; FWST, falling when standing.

### 5.2 Experiment 2: precision, recall and F1 score

In this experiment, the proposed system undergoes a thorough evaluation, accompanied by an in-depth analysis of its specific implications in certain domains. Table 7 presents the evaluation matrics, including precision, recall and F1 score for both datasets.

**TABLE 7:** Precision, Recall, and F1 score for ADL and Fall activities over UMA_fall and UR-fall Datasets.

Classes	UMA_fall ADL activities			UMA_ fall Fall activities			UR-fall ADL activities			UR-fall fall activities		
Activities	Precision	Recall	F1 score	Precision	Recall	F1 score	Precisiom	Recall	F1 score	Precision	Recall	F1 score
BD	99	97	98	—	—	—	—	—	—	—	—	—
CD	96	97	97	—	—	—	—	—	—	—	—	—
CU	96	97	97	—	—	—	—	—	—	—	—	—
HP	97	96	96	—	—	—	—	—	—	—	—	—
JG	97	97	97	—	—	—	—	—	—	—	—	—
LD	98	96	97	—	—	—	—	—	—	—	—	—
SD	96	97	97	—	—	—	—	—	—	—	—	—
WK	96	97	97	—	—	—	—	—	—	—	—	—
Fall forward	—	—	—	95	95	95	—	—	—	—	—	—
Fall backward	—	—	—	95	94	94	—	—	—	—	—	—
Fall lateral	—	—	—	94	95	95	—	—	—	—	—	—
ST	—	—	—	—	—	—	94	95	95	—	—	—
SI	—	—	—	—	—	—	93	95	94	—	—	—
LD	—	—	—	—	—	—	92	94	93	—	—	—
BD	—	—	—	—	—	—	96	92	94	—	—	—
CR	—	—	—	—	—	—	95	94	95	—	—	—
WLK	—	—	—	—	—	—	95	94	95	—	—	—
PR	—	—	—	—	—	—	94	95	95	—	—	—
OT	—	—	—	—	—	—	96	92	94	—	—	—
Fall forward	—	—	—	—	—	—	—	—	—	93	92	93
Fall backward	—	—	—	—	—	—	—	—	—	91	91	91
Getup (from chair)	—	—	—	—	—	—	—	—	—	89	92	91
FWS	—	—	—	—	—	—	—	—	—	93	92	93
FWST	—	—	—	—	—	—	—	—	—	94	93	94

#### 5.2.1 Discussion and analysis

The analysis of the fall detection and activity recognition performance using the UMA_Fall and UR_Fall datasets demonstrates strong reliability across various activities. The precision, defined as the rate of accurate identification of activities, is notably high for some activities such as ‘Lying Down,’ ‘Jogging,’ and ‘Sitting Down’ in the UMA_Fall dataset, exceeding 96. This indicates the reliability of the method for monitoring essential everyday activities in elder care and rehabilitation. Similarly, activities that resemble falling, such as ‘Fall Forward,’ ‘Fall Backward,’ and ‘Fall Lateral,’ exhibit exceptionally high precision and recall, specifically 95, so affirming the adequacy of the proposed model for real-time fall detection. In the context of the UR_Fall dataset, the ‘Standing’ and ‘Sitting’ categories exhibit precision and recall exceeding 93, indicating the model’s adaptability across various sensors. Nonetheless, certain movement transitions exhibit marginally reduced accuracy, as evidenced by a recall of 89 for the state ‘Get up (from chair)' in the UR_Fall dataset; this aspect could be improved to better capture significant, albeit occasionally subtle, movements for various applications, such as physical therapy or workplace ergonomics. The F1 score, which measures the average of precision and recall, remained elevated in most tasks, reflecting the model’s overall competence. Notable favorable connections exist between health monitoring and fall prevention, while there are places where the system’s effectiveness regarding activity transitions might be enhanced.

A significant factor in the detection of falls is false positives caused by high-velocity ADLs like sudden sitting or lying down. Such actions can cause motion signatures that are very similar to falls when using only accelerometer or gyroscope signals. Our system’s sensor fusion, however, through visual and inertial features like body posture modeling, curve dynamics, and 2.5D point cloud transition allows successful discrimination to be performed. As exemplified by the confusion matrices (Tables 4 and 5), activities like “Getup (chair)” and “Sitting” are differentiated well from fall events, verifying the learning system’s capability to break down motion similarities through semantic and context clues.

### 5.3 Experiment 3: ROC (receiver operating characteristic curve)

**FIGURE 16:**
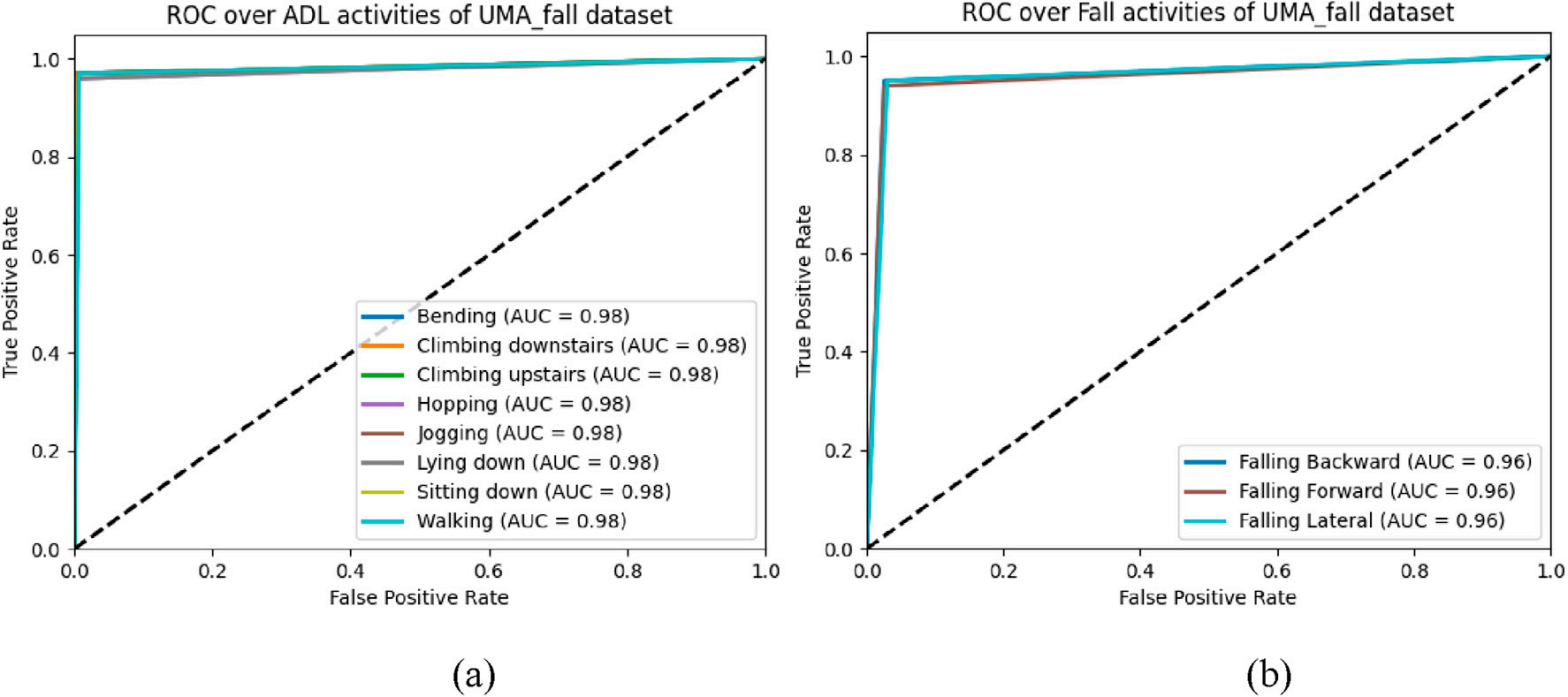
ROC curves for the UMA_Fall dataset: **(a)** ADL activities, **(b)** Fall activities.

The Receiver Operating Characteristic (ROC) curves presented in Figures 16a,b depict the performance of a categorization system of various health related activities. The AUC under the ROC curve provides a single metric summarizing the performance. The closer the AUC gets to 1, the better the model is at distinguishing the positive class (the specific activity) from the negative class (all the other activities).

#### 5.3.1 Discussion and analysis of ROC curve

In Figure 16a, by considering the ADL dataset of the UMA_fall dataset, it is clear that the ‘Walking’ and ‘Sitting’ activities demonstrated the maximum model efficiency with approximately 100 percent accuracy as seen by the AUC values equal to 0.98. The other movements, such as ‘Climbing downstairs,’ ‘Bending,’ ‘Climbing upstairs,’ ‘Hopping,’ ‘Jogging,’ and ‘Lying down,’ also exhibit remarkable model performance with an AUC of 0.98 for all. These outcomes illustrate the capacity of the model to recognize a large variety of ADLs utilizing a decreased quantity of misclassification. In Figure 16b, which examines fall-related activities, the model likewise performs robustly, reaching AUCs of 0.96 across all three fall scenarios: These include ‘Falling Backward, Falling Forward’, and ‘Falling Lateral’. This is significant in fall detection systems and shows that the suggested model is capable of identifying falls with sufficient reliability across the different types of falls. The AUCs, albeit high, are slightly lower than those obtained by the identical activities in ADLs, which hint to the prospective opportunity for development in dealing with more sophisticated fall detection circumstances.

### 5.4 Experiment 5: comparisons with state of the art (SOTA)

In (Modak et al., 2024), the UMA_Fall dataset, with its 25 hand-engineered features, enabled the delopment of hybrid models combining 1D CNNs and classifiers like Xception and SVM, achieving 92% accuracy. Its sensor-based approach is effective for mobility applications but poses compliance challenges, especially for elderly users. (Hafeez et al., 2023). integrated inertial sensors and RGB features, combining accelerometer data with skeletal tracking from Kinect to achieve high accuracy in ADL and fall detection. However, synchronization challenges between sensors remain. Another study used the URFall datasets, leveraging histogram and motion vector features to achieve 92.83% accuracy, respectively. While highly accurate, distinguishing between similar activities remains an issue.

In (Nooyimsai et al., 2022), a CNN-LSTM ensemble model classified falls into non-fall, pre-fall, and fall states using UMA_Fall dataset, achieving state-of-the-art accuracies of 96.16%. The approach combined temporal modeling and feature extraction, demonstrating strong real-world potential. Finally, a novel macro-feature-based method in (Beddiar et al., 2022) utilized Le2i and UR-FD datasets to calculate body posture angles and distances, achieving high performance with LSTM, TCN, and SVM models, though improvements in annotation and posture differentiation are needed. Together, these works demonstrate the strengths of sensor-based (e.g., UMA_Fall and UR-Fall) datasets. Combining these approaches through hybrid systems, such as multi-sensor fusion and CNN-LSTM ensembles, could enhance robustness, accuracy, and applicability in diverse fall detection scenarios. Table 8 shows the comparison of proposed method with state-of-the-art methods.

**TABLE 8:** Comparisons with state of the art.

Method	Accuracy %	
	UMA_fall	UR-fall
Modak et al. (2024)	92	—
Nooyimsai et al. (2022)	82.24	—
Kabir et al. (2023)	96.12	—
Merrouche and Baha (2020)		89.00
Beddiar et al. (2022)		85.00
Hafeez et al. (2023)		92.83
Proposed	97	94

## 6 Implication of proposed system

There are many implications of the proposed system since the strong performance of the metrics for both the UMA_Fall and UR_Fall datasets. The AUC of ROC curves show that the algorithm performs well for ‘Walking’ and ‘Sitting’ as well as falling activities such as ‘Falling Backward, Falling Forward,’ and ‘Falling Lateral.’ From these results, it can be concluded that the system indeed possesses an excellent true positive detection rate in both the daily actions and autumn events with low false positive rates of the algorithm. Precision, recall and F1 score are also used to give more information about the reliability of the system. In the UMA_Fall dataset, the ‘Lying Down’ F1 score is 97, ‘Jogging’ F1 score is 97, and ‘Sitting Down’ F1 score is 97, it shows that the proposed model works effectively in accurate classification of regular movements. Typical fall actions such as “fall forward,” “fall backward,” and “fall laterally” reliably achieve F1 scores of 95, illustrating the system’s resilience in principal fall scenarios. With the UR_Fall dataset, the basic activities – ‘Standing’ (F1 = 95) and ‘Walking’ ((F1 = 95) demonstrate that the system functions well with multiple sensors; ‘Get up (from chair)’ results demonstrated somewhat lower accuracy (F1 = 91) which could be fine-tuned. Such findings clearly suggest the possibility of the use of the system in a myriad of fields such as elder care, emergency response, and smart homes among others.

## 7 Conclusion

This work introduced a new and extremely efficient paradigm for human action identification based on information fusion from RGB and inertial sensors, state-of-the-art machine learning methods, and DNN. The proposed system achieves high accuracy in distinguishing ADLs and falls using optimized features: 2.5D point cloud, kinetic energy, and inertial coefficients. Higher levels of preprocessing and accurate segmentation improved the quality and trustworthiness of features in various datasets. The performance and flexibility given by the system recommend it as a suitable option for long-term health checks and fall detection in aged care and practical healthcare settings.

Evaluations on the UMA_Fall and UR-Fall datasets showcased the system’s ability to generalize effectively, achieving high classification accuracies and demonstrating robustness to variations in working conditions such as sensor placement, participant diversity, and environmental noise. Techniques such as regularization, data augmentation, and cross-validation ensured the stability and reliability of the method across different scenarios.

The performance and flexibility offered by the system make it a promising option for long-term health monitoring and fall detection, particularly in elderly care and practical healthcare applications. By leveraging advanced multimodal data fusion and robust feature extraction, the system provides a dependable solution for real-world healthcare challenges, paving the way for improved patient outcomes and proactive health management.

## Data Availability

Publicly available datasets were analyzed in this study. This data can be found here: https://paperswithcode.com/dataset/urfd-dataset, https://figshare.com/articles/dataset/UMA_ADL_FALL_Dataset_zip/4214283.
